# Health-Promoting and Functional Properties of Fermented Milk Beverages with Probiotic Bacteria in the Prevention of Civilization Diseases

**DOI:** 10.3390/nu17010009

**Published:** 2024-12-24

**Authors:** Milena Alicja Stachelska, Piotr Karpiński, Bartosz Kruszewski

**Affiliations:** 1 Faculty of Health Sciences, University of Lomza, Akademicka 14, 18-400 Łomża, Poland; pkarpinski@al.edu.pl; 2 Department of Food Technology and Assessment, Institute of Food Sciences, Warsaw University of Life Sciences—SGGW, Nowoursynowska 159 C, 02-776 Warsaw, Poland

**Keywords:** lactic acid bacteria, probiotic bacteria, obesity, lactose intolerance, diabetes, cancer protection, civilization diseases, intestinal microbiota, intestinal pathogens

## Abstract

Background/Objectives: There is scattered information in the scientific literature regarding the characterization of probiotic bacteria found in fermented milk beverages and the beneficial effects of probiotic bacteria on human health. Our objective was to gather the available information on the use of probiotic bacteria in the prevention of civilization diseases, with a special focus on the prevention of obesity, diabetes, and cancer. Methods: We carried out a literature review including the following keywords, either individually or collectively: lactic acid bacteria; probiotic bacteria; obesity; lactose intolerance; diabetes; cancer protection; civilization diseases; intestinal microbiota; intestinal pathogens. Results: This review summarizes the current state of knowledge on the use of probiotic bacteria in the prevention of civilization diseases. Probiotic bacteria are a set of living microorganisms that, when administered in adequate amounts, exert a beneficial effect on the health of the host and allow for the renewal of the correct quantitative and qualitative composition of the microbiota. Probiotic bacteria favorably modify the composition of the intestinal microbiota, inhibit the development of intestinal pathogens, prevent constipation, strengthen the immune system, and reduce symptoms of lactose intolerance. As fermented milk beverages are an excellent source of probiotic bacteria, their regular consumption can be a strong point in the prevention of various types of civilization diseases. Conclusions: The presence of lactic acid bacteria, including probiotic bacteria in fermented milk beverages, reduces the incidence of obesity and diabetes and serves as a tool in the prevention of cancer diseases.

## 1. Introduction

Fermented milk beverages are produced based on lactic acid fermentation, which, at the same time, is a method of milk preservation. The classification of this group of products is based on the type of basic microflora used in the production of fermented milk beverages, which is divided into thermophilic and mesophilic due to the optimal temperature of their growth. The typical thermophilic bacteria are *Lactobacillus delbrueckii* ssp. *bulgaricus* and *Streptococcus salivarius* ssp. *thermophilus*. These are the typical starter microflora used in the production of yogurt. For the production of kefir, in addition to mesophilic bacterial species, yeast is used, and then the production of fermented milk beverages is carried out through mixed lacto-alcohol fermentation [1]. Products formed as a result of lactic fermentation are characterized by a pure sour taste, given by lactic acid, and enriched by the main flavor components in the form of diacetyl and acetaldehyde [2]. When using mixed starters, which contain yeast in addition to lactic fermentation bacteria, used for the production of kefir, the additionally formed ethyl alcohol and carbon dioxide give a specific, slightly sharp, yeasty flavor. Fermented dairy beverages have a special nutritional importance. They are valued not only for their great palatability but also for their unique nutritional, preventive, and medicinal properties [3].

The main dairy products made with pure bacterial cultures include traditional fermented beverages such as yogurt, kefir, acidophilic milk, and new-generation dairy products. The first of them is produced with selected lactic acid bacteria, while the second group uses bacterial strains isolated from the digestive tract of healthy humans, mainly *Bifidobacterium*. Among the new generation of dairy products, bio-yogurts, which are popular in the Polish market, deserve special attention. Both of these groups of dairy products have specific effects on our body [4]. The lactic acid bacteria present in traditional dairy beverages are very sensitive, and most of them die in the digestive tract due to the bactericidal effect of gastric juice. Bacteria introduced with new-generation beverages easily overcome the acidic stomach environment by settling in the mucous membranes of the digestive tract. There, their rapid multiplication takes place, which has an extremely beneficial effect on the state of our internal bacterial flora [5].

Fermented milk beverages are an extremely valuable source of nutrients with high nutritional and biological value. They contain high-quality proteins with a balanced amino acid composition and are also a source of easily digestible calcium, phosphorus, and potassium [6]. These products are characterized by very high digestibility. Bacterial enzymes contained in these products cause the initial digestion of proteins and the release of free amino acids that are essential for our body. The presence of lactic acid contributes to the degradation of the main protein of milk-casein in the form of small particles, which significantly facilitates the digestion process. The digestion of fat also increases as a result of bacterial enzymes. For these reasons, these products are especially recommended for the elderly and people with gastrointestinal ailments. Fermented beverages contain substances with antibacterial properties that inhibit the growth and activity of putrefactive bacteria. In addition, the microorganisms contained in milk contribute to the additional biosynthesis of important vitamins of the B group for our body [7].

The biological value of food is closely related to the nutrient content in the food and its bioavailability. Fermented milk beverages contain similar amounts of nutrients that are in the milk from which they are made. However, due to the processes of lactic fermentation, they are better absorbed by the human body in the digestive process. Milk proteins are broken down into simpler and more easily digested compounds in the form of low-molecular-weight peptides and free amino acids. Lactic acid bacteria produce lipolytic enzymes that break down fats to glycerol and free fatty acids. Lipolysis processes help improve the digestibility of milk fat, which is present in fermented milk beverages [3].

The best source of probiotic bacteria is fermented milk beverages (yogurt, kefir, and acidophilus milk). Probiotic bacteria are primarily used in the dairy industry as starter cultures to carry out acid coagulation, i.e., the formation of curd in the manufacturing of yogurt or kefir. The metabolic products of probiotic microorganisms give fermented milk beverages their health-promoting properties and shape the appropriate organoleptic characteristics. The term “probiotic” comes from the Greek “pro bios”, meaning “for life”. The current definition of a probiotic is provided by the World Health Organization (WHO). According to it, probiotics constitute specific species that, when provided to humans (or animals) in an appropriate dose, exert a beneficial effect on the organisms. They are well-defined microorganisms that help improve the balance of the microflora inhabiting different areas of the human body and thus have a beneficial health effect. Probiotic microorganisms are lactic acid bacteria, meaning they produce lactic acid and mainly belong to the genera *Lactobacillus* and *Bifidobacterium*. The most important representatives of probiotic bacteria with documented health-promoting properties are *Lactobacillus acidophilus*, *Lacticaseibacillus rhamnosus*, and *Lactobacillus delbrueckii* ssp. *bulgaricus*, *Lactiplantibacillus plantarum*, *Lacticaseibacillus casei* and *Bifidobacterium bifidum* [5].

The probiotic microflora that primarily inhabits our digestive system is constantly being destroyed due to the overuse of antibiotics, consumption of processed foods, acid-forming products, food preservatives, alcohol, and some medications, which can lead to intestinal imbalance. Probiotics, by colonizing and multiplying in our large intestine, ensure the maintenance of the proper balance of intestinal microbiota and the body’s natural balance by having a beneficial effect on our digestive tract. The human digestive system is also inhabited by undesirable microorganisms, including pathogenic ones, whereby probiotic bacteria inhabiting our digestive tract in large numbers defend our body from the action of unfavorable bacteria. They inhibit the growth of unfavorable bacteria in the intestine. They produce bacteriostatic substances, as well as bactericidal substances, including lactic acid, which leads to a lowering of the pH in the intestines, and thus, many pathogenic microorganisms are killed. Consumption of more and more highly processed foods (fast food), often containing too much fat and not enough vegetables, is also a factor that adversely modifies the composition of human gut microorganisms. Probiotic bacteria play a huge role in the prevention of civilization diseases and aging and contribute to the overall improvement of human health. Probiotic bacteria favorably modify the composition of the intestinal microbiota, inhibit the development of intestinal pathogens, protect against cancer, lower bad cholesterol, prevent constipation, strengthen the immunological system, decrease the occurrence of obesity, and reduce symptoms of lactose intolerance [8,9,10].

There is scattered information in the scientific literature regarding the characterization of probiotic bacteria found in fermented milk beverages and their beneficial effects on human health. The objective of this review was to gather available information on the use of probiotic bacteria in the prevention of civilization diseases, with a special focus on the properties of probiotic bacteria in the prevention of obesity, diabetes, and cancer.

## 2. Modification of the Composition of the Intestinal Microbiota

The term “microbiota” should be understood as all microorganisms, i.e., bacteria, fungi, eukaryotes, and viruses that populate the human body. It is known that up to 1014 different bacteria make up the entire human microbiota. Also, it is known that 70% of the microflora inhabits the large intestine. The role of the microflora relies on protection against infections, digestion of long-chain carbohydrates and dietary fibers, production of short-chain fatty acids and synthesis of vitamins, as well as regulation of the immune response and metabolic balance. Microbiota imbalances are at the root of many diseases, including allergies, diabetes, obesity, inflammatory bowel disease, functional gastrointestinal diseases, neurodegenerative diseases, depression, and autism. Thus, consuming fermented milk beverages containing strains of probiotic bacteria can help prevent many diseases. The intestinal microbiota can be effectively modified through the use of probiotics, prebiotics, and synbiotics.

The results of recent studies have shown that the development of the gut microbiota begins already during prenatal life. Still, intensive colonization of the gastrointestinal tract occurs at the time of delivery, during exposure to bacteria of the birth tract (in the case of natural childbirth), or to bacteria residing in the skin area if delivery is by cesarean section [11]. The mode of feeding during the neonatal period is also important for regulating the composition of the microbiota, with a very significant advantage in the case of natural feeding with breast milk, which, in addition to its nutritional value, contains proteins with antibacterial properties against pathogens, secretory immunoglobulin A, bacteria (in naturally fed children, *Bifidobacterium* and *Lactobacillus* predominate in the gastrointestinal tract) and oligosaccharides with prebiotic effects. The latter promotes the growth of commensal bacteria and prevents the colonization of the gastrointestinal tract by pathogens [12,13]. However, an infant’s microbiota is highly unstable and characterized by low microbial diversity.

The physiological role of the microbiota is very broad. It includes protection against gastrointestinal infections, maturation of the immune system, regulation of host metabolic pathways, digestion of long-chain carbohydrates or dietary fiber, as well as the formation of short-chain fatty acids and vitamins [14,15]. However, in light of recent studies, the impact of intestinal microbiota on such functions of the central nervous system as the formation of cognitive functions and basic behavioral patterns, social interactions, or coping with stress should also be emphasized with full emphasis. In view of the important role played by the microbiota, the term “bacterial organ” given to it seems appropriate. This organ is a component of the brain-gut-microbiota axis, which structurally includes the central nervous system, the neuroendocrine and neuroimmune systems, the parasympathetic and sympathetic parts of the autonomic nervous system, the visceral nervous system, and, most importantly, the microbiota. The components of the axis maintain a two-way communication of signal networks; those from the brain affect motility, sensation, and secretion, while visceral signals from the gut exert influence on central functions [16,17,18].

The term “dysbiosis” refers to disorders in the composition, proportion, and function of the microbes that make up the microbiota. The etiopathogenesis of many diseases is linked to disorders of the microbiota, although in most cases, they are not the sole etiological factor. The specific “pattern” of microbiota disorders attributed to a given disease entity has not been clearly established. However, dysbiosis is known to play a role in the pathogenesis of inflammatory bowel disease (IBD), functional gastrointestinal diseases headed by irritable bowel syndrome, allergic diseases, diabetes and obesity, steatohepatic disease, atherosclerosis, as well as in neurodegenerative diseases, depression, autism, and schizophrenia [19,20,21,22]. The most consistent feature accompanying diseases is lower diversity of the microbiota, as it is known that genetic diversity of the microbiome is an important factor affecting the health of the host organism. For many decades, the belief prevailed that the composition and structure of the microbiota in a given host is constant from childhood to old age. In light of the results of modern research, it is known that it can undergo changes, including those going in the direction of dysbiosis, which can be led by a great many factors. They include diet, infectious diseases, the use of antibiotic therapy and drugs such as proton pump inhibitors or non-steroidal anti-inflammatory drugs, and the level of hygiene in a given environment or exposure to stressful situations [23,24].

Recognition of the role of the microbiota in the maintenance of homeostasis and its disorder in the pathogenesis of many diseases has led to tremendous interest in methods of modification. Among the very important methods for modifying the composition of the intestinal microbiota is the use of probiotics and prebiotics. Probiotics are live microorganisms that, when administered in adequate amounts, exert a beneficial health effect on the host body. Prebiotics, per contra, are food elements that are not digested but selectively stimulate the growth or metabolic activity of specific beneficial bacteria in the large intestine and thus have a beneficial effect on human health. Synbiotics consist of probiotics with prebiotics used together. The pursuit of beneficial modification of the intestinal microbiota as a prophylaxis or treatment of many diseases has generated tremendous interest in the potential use of probiotics. The definition of probiotics given above was developed in 2001 and confirmed by the International Scientific Association for Probiotics and Prebiotics (ISAPP) [25].

When selecting the use of a probiotic preparation, it is very important to know the taxonomic identification of microorganisms and to know that each of them should be identified not only by genus and species but also by strain. This is because the properties of probiotics are strictly strain-dependent, and each strain requires separate studies to determine its properties and efficacy in a specific clinical situation. The results of studies conducted on one specific strain and at a specific dose cannot then be extrapolated to other related but untested strains and doses [26,27]. Thus, in characterizing the “ideal” optimal probiotic strain, it should be emphasized that it is of human origin, non-pathogenic, resistant to gastric juice, pancreatic enzymes, and bile acids, survivable and active despite technological and manufacturing processes, able to adhere to intestinal mucosal epithelial cells, antagonistic to pathogens, beneficial to host metabolism and, as emphasized above, clinically tested.

In addition to single-strain probiotic preparations, there are also multi-strain preparations, the clinical effects of which should also be evaluated in a reliable clinical trial since the effects of the joint action of individual strains can be both synergistic and antagonistic [26]. At this point, it should also be mentioned that it is currently impossible to give general rules for the dosage of probiotics, and it is appropriate to apply such a dose that in a reliable study in humans, using the necessary strain for implementation showed a beneficial effect [27]. The mechanisms of action of probiotics are still the subject of research and discussion. It should be noted that it is not possible to consider a single mechanism of action common to all probiotic strains. ISAAP experts have distinguished three main directions of probiotic activity: (1) mechanisms that are common and shared by many types of probiotics, such as the production of short-chain fatty acids; (2) common mechanisms shared by individual species, such as the metabolism of bile acid salts; and (3) rare mechanisms specific only to individual strains (e.g., production of bioactive factors, endocrine and neurogenic effects, effects on the immune response) [26].

Clinically effective, i.e., beneficial modification of the microbiota with probiotics in the light of evidence-based medicine, as shown by analysis of randomized trials or their meta-analysis in recent years, has been shown to treat acute infectious diarrhea in children and (according to the World Gastroenterology Organization [WGO]) also in adults, reducing diarrhea by one day [26,27,28] and preventing diarrhea associated with antibiotic use in both adults and children [29,30]. In the latter indication, the role of *Saccharomyces boulardii* and *Lactobacillus* GG is best documented [31,32,33]. For diarrhea caused by *Clostridium difficile* infection, which accounts for up to 25% of antibiotic-associated diarrhea episodes, the results of several meta-analyses have shown that the prophylactic use of probiotics (evaluated together) reduces the risk of diarrhea caused by this infection, with an emphasis on probiotic use within the first 2 days of antibiotic therapy [34,35].

Among the potentially effective strains are *Bifidobacterium infantis* 35,624 and *L. plantarum* 299v, and in children, *Lactobacillus* GG [36,37,38]. Probiotic therapy has been evaluated in clinical trials among patients with IBD. While their efficacy in Crohn’s disease has not been confirmed, in moderate and mild forms of ulcerative colitis, the addition of probiotics can achieve a faster response and maintain remission than conventional therapy alone. Beneficial effects have also been obtained in moderate intestinal reservoir inflammation after colectomy and prevention of recurrence of this inflammation [39]. According to the Food and Drug Administration (FDA), which has granted probiotics “generally recognized as safe” (GRAS, generally recognized as safe) status, they are safe and well tolerated. In European Union countries, probiotics have been granted “qualified presumption of safety” (QPS) status. However, caution in administration and consideration of the appropriateness of use should be exercised in patients with immunodeficiencies, in severe clinical conditions, and treated in intensive care units.

## 3. Reducing the Risk of Obesity

Obesity is a disease that results from excessive body fat (the body’s natural store of excess energy stored in it). Thus, it is a chronic disease with no tendency to subside on its own. Its occurrence in the modern world is steadily increasing, and it affects children, adolescents, and adults alike. It has been shown that fermented milk products can be utilized as adjuvant care in order to decrease total cholesterol, LDL cholesterol, and triglycerides in the blood. Yogurt may significantly contribute to the reduction of weight because it contains probiotics, has a high protein and calcium content, and has a low glycemic index [9]. There are many reasons for the occurrence of obesity, but the most common causes are environmental factors, including excess food intake and too little physical activity. Endocrine, metabolic, and psychological disorders (such as depression) and genetic factors are also among the causes. However, all of these factors provide no explanation for the magnitude of disorders affecting human metabolism. Increasingly, the scientific world is paying attention to the involvement of the microbiota in metabolic and energy disorders. Ongoing research has already established the role of the intestinal microbiota in the process of digestion and obtaining energy from food. It is known that gut bacteria are proven to be responsible for the growth as well as differentiation of intestinal epithelium, fermentation of undigested nutrients, synthesis of B vitamins and vitamin K as well as amino acids (such as lysine and threonine), metabolism of dietary cholesterol and bile acids, and absorption of micronutrients [40].

However, the question still remains as to what composition of bacteria in the gastrointestinal tract protects it and what promotes metabolic disorders. Indeed, each person has its own unique diversity of the intestinal microbiota ecosystem. This ecosystem includes about 1000 phylotypes (weighing 1–1.5 kg), which constitute different taxonomic units: strains, species, and genera. Previous experimental studies to assess the trophic function of the microbiota have been conducted on gnotobiotic germ-free (free of detectable microorganisms and parasites) mice. Transplantation of gut bacteria from obese mice or from obese individuals resulted in rapid obesity and insulin resistance in germ-free mice [41]. This is explained by more efficient absorption of energy from food under the influence of the transplanted microbiota and a higher content of energy-rich short-chain fatty acids in their digestive tract [42]. One study has shown that some bacterial strains have the ability to synthesize glycoside hydrolases, enzymes necessary to break down plant polysaccharides previously considered impossible to digest in the human body (such as insoluble fiber). This process can increase the energy amount provided to the host by 80–200 kcal (i.e., 4–10% of 24-h energy) [40]. It has also been found that through hydrolysis and fermentation of short-chain fatty acids (propionic acid, acetic acid, and butyric acid) and following their absorption, microbiota can increase the host’s energy intake by 150 kcal/day. Based on other studies, it has been shown that short-chain fatty acids can affect host appetite through the Gpr41 receptor, GLP-1 secretion, and the FFAR2 receptor. It also alters intestinal passage time or energy recovery and utilization, and this translates into increased human energy balance [43]. Fatty acid SCFAs also affect G proteins produced by gut endocrine cells, which stimulate the release of the molecular appetite controllers GLP-1 (glucagon-like peptide-1) and peptide YY. Peptide YY is a hormone that has been of particular interest to researchers in recent years. This is because it decreases intestinal motility and slows intestinal passage, and thus may increase nutrient absorption time and the delivery of energy value to the host [44].

Information that the intestinal microbiota is differentiated in slim and obese people has prompted research into the use of targeted bacterial genera and strains for the prevention and treatment of obesity and comorbidities. The genera *Lactobacillus* and *Bifidobacterium*, in particular, have been shown to have beneficial effects in this regard. This is because they reduce the intensity of inflammation in the intestine, improve the efficiency of the intestinal barrier, and reduce, as well as, what is most important, maintain reduced body weight. *Bifidobacterium* has been found to increase the expression of genes encoding the proteins of tight junctions between enterocytes [45,46]. In contrast, *Lactobacillus* has been found to reduce visceral fat and adipocyte size [47,48,49], improve insulin sensitivity, affect leptin secretion and the synthesis of fatty acids, as well as inhibit lipoprotein lipase activity [50,51]. In an intervention study, a probiotic containing *Lactobacillus gasseri SBT2055* was administered to patients for 12 weeks. In overweight patients, it caused a significant reduction in their body weight, as well as a reduction in subcutaneous and visceral fat, BMI (body mass index), waist and hip circumference, and on top of that, adiponectin levels increased in their blood [52]. Obese patients, on the other hand, achieved reductions in weight, BMI, waist and hip circumference, and body fat [53]. Meanwhile, in a randomized, double-blind study, a strain of *L. rhamnosus CGMCC1.3724* was administered to obese women and resulted in a reduction in body weight, body fat, and serum leptin levels [54].

People who drank the most whole-fat yogurt were significantly less prone to develop metabolic syndrome components such as abdominal obesity, hypertriglyceridemia, low HDL cholesterol, high blood pressure, and high fasting plasma glucose, according to Babio et al. [55]. Yogurt consumption was strongly associated with a lower chance of gaining weight, according to a study that examined three large cohorts of people for up to 20 years and included 120,877 obese men and women free of chronic conditions at the start of the trial. The authors concluded that the bacteria that live in the digestive tract could be responsible for observed variations in weight [9]. The promising therapeutic effects of probiotic yogurt consumption in humans in the treatment of obesity are presented in Table 1.

Prebiotics should also be considered in the therapy of conditions such as overweight and obesity. Their primary function is to selectively stimulate the growth and activity of selected types of intestinal microbiota. In experiments on animal models, prebiotics, i.e., inulin and fructooligosaccharides, stimulated the growth of *Bifidobacteria*, *Bacteroidetes*, *Prevotella*, and *Roseburia* and decreased the population of *Firmicutes* [56,57]. In contrast, after inulin was used for 3 months in obese women, beneficial changes in the intestinal ecosystem were observed in the form of an increase in *Bifidobacterium* and *Faecalibacterium prausnitzii* and a decrease in *Bacteroidetes* and *Propionibacterium*. Probiotics administration usually has positive effects on reducing weight in people. However, some studies have shown contrasting results. Different *Lactobacillus* species are connected with various effects on weight modification that are host-specific. *L. acidophilus* administration resulted in significant weight gain in humans and animals (SMD 0.15; 95% confidence intervals 0.05–0.25). Results were consistent in humans and animals. *Lactobacillus fermentum* and *Lactobacillus ingluviei* were associated with weight gain in animals. *L. plantarum* was associated with weight loss in animals, and *L. gasseri* was associated with weight loss both in obese humans and in animals [58]. What is more, ready-for-consumption remedial food containing probiotic organisms gave similar results for weight gain and the treatment of malnutrition compared to conventional remedial food without probiotics. As an effect, early shaping of gut microbiota composition through the management of probiotics presumably results in raised weight gain [59].

**Table 1:** Promising therapeutic effects of probiotics consumption in the treatment of human obesity.

Activity	Effect	References
Reduction of body weight	In an intervention study, a probiotic containing *L. gasseri SBT2055* was administered to patients for 12 weeks. In overweight patients, it caused a significant reduction in their body weight, as well as a reduction in subcutaneous and visceral fat, BMI, waist and hip circumference, and on top of that, adiponectin levels increased in their blood.	[52]
Reduction of body weight	Obese patients achieved reductions in weight, BMI, waist and hip circumference, and body fat.	[53]
Reduction of body weight	The strain of *L. rhamnosus* CGMCC1.3724 was administered to obese women and caused a reduction of body weight, body fat, and serum leptin levels.	[54]
Prevention of abdominal obesity and accompanying symptoms	Drinking whole-fat yogurt significantly decreased the risk of development of metabolic syndrome factors like abdominal obesity, high blood pressure, hypertriglyceridemia, high fasting plasma glucose, or low HDL cholesterol.	[55]
Prevention of gaining weight	Yogurt drinking was strongly associated with a lower possibility of increasing weight in people not exceeding 20 years with obesity but without chronic conditions at the start of the tests.	[9]
Weight loss in overweight and obese populations	Consumption of probiotics or synbiotics can cause significant weight decreases, either by maintaining usual lifestyle habits or together with energy restriction and/or enhanced physical activity for a period of 12 weeks. Strains within the genera *Lactobacillus* and *Bifidobacterium* were the most utilized and exhibited the best results in reducing body weight.	[60]
Reduction of body weight	Results showed that the probiotic supplementation was significantly effective in reducing weight. Better effects on BW occurred when the duration of supplementation was higher than 8 weeks and on obese persons. BMI was significantly impacted as well in participants with metabolic syndrome and when the supplementation period lasted for more than 12 weeks.	[61]
No significant effect of probiotics on weight and BMI after bariatric surgery (BS)	The possibility of probiotics usage as an accompaniment to bariatric surgery (BS) is disputable. Results showed a statistically significant difference during probiotics consumption in the decrease in waist circumference at 12 months after BS. However, probiotics were not effective in weight and BMI both within 3 months and at 12 months postoperation. Probiotics help people with morbid obesity in order to achieve waist circumference improvement after BS, with no impact on weight and BMI.	[62]
No significant effects on birth weight or weight gain during pregnancy and infancy	Probiotics and synbiotics did not demonstrate any profitable result on weight and BMI in pregnant women by 6.6 × 10^5^–6.6 × 10^10^ CFU/day of probiotics during 1–25 weeks and 1 × 10^9^–112.5 × 10^9^ CFU/capsule of synbiotics during 4–8 weeks. The result of biotics on weight and BMI in the case of infants was non-significant. Prebiotics and probiotics used in infancy were from 0.15 to 0.8 g/dL and 2 × 10^6^–6 × 10^9^ CFU/day for 2–24 weeks, respectively. They had no significant effects on birth weight or weight gain during pregnancy and infancy.	[63]
Significant weight gain as well as weight loss in humans and animals	*L. acidophilus* management emerged in meaningful weight gain in both humans and animals. Effects were coherent in humans and animals. *L. fermentum* and *L. ingluviei* were connected with weight gain in animals. *L. plantarum* was connected with weight loss in animals. *L. gasseri* was linked with weight loss in all subjects.	[58]
Weight gain in children with malnutrition, weight loss in people with obesity	Revised knowledge about the capacity of specific probiotics to gain energy from the host diet might introduce the development of new therapies for obesity and malnutrition. Probiotics and antibiotics contribute to weight gain or weight loss in human beings. Ready-to-use remedial food led to weight gain in children and limited the commonness of malnutrition.	[59]

In other studies, prebiotics improved appetite control by affecting the stimulation of GLP-1 and peptide YY secretion and reducing the production of ghrelin, thus contributing to the reduction of body weight, adipocyte size, and the amount of adipose tissue. Moreover, they counteracted the accumulation of fatty acids in the liver, which may prevent liver steatosis [64,65]. In contrast, in obese women with dyslipidemia, the use of oligofructose for 120 days reduced body weight, BMI, and waist circumference and lowered serum total cholesterol and LDL fractions [66]. Prebiotics (oligofructose, arabi-noxylose) have also been credited with reducing intestinal inflammation, improving intestinal barrier integrity, stimulating GLP-2 secretion, and improving glucose tolerance [67]. Both probiotics and synbiotics have the prospective implications of aiding in weight reduction for overweight and obese populations [60]. The findings demonstrated that probiotic supplementation was significantly effective in decreasing weight [61]. It seems that probiotic products could have beneficial effects as an adjunct therapy for the care and management of obesity when used in high doses [68]. Another study demonstrates that oral probiotics supplementation can be a complementary treatment for improving the fitness of postmenopausal women with overweight and obesity [69]. Probiotics are effective in generating body weight loss in patients who are overweight or obese with connected metabolic diseases [70,71,72]. However, more quality clinical studies are needed to confirm the efficacy and safety of probiotics and address a number of practical issues before the routine clinical use of probiotics in adults with obesity undergoing BS [62]. Some studies showed that probiotics and synbiotics did not demonstrate any beneficial effect on weight and body mass index (BMI) in pregnant women by 6.6 × 10^5^–6.6 × 10^10^ CFU/day of probiotics during 1–25 weeks and 1 × 10^9^–112.5 × 10^9^ CFU/capsule of synbiotics during 4–8 weeks. The effect of biotics on weight and BMI in infants was predominantly non-significant. Prebiotics and probiotics used in infancy were from 0.15 to 0.8 g/dL and 2 × 10^6^–6 × 10^9^ CFU/day for 2–24 weeks, respectively. They had no significant effects on birth weight or weight gain during pregnancy and infancy [63]. In the treatment of obesity and metabolic disorders, it is worthwhile to use probiotics or live bacteria, which have beneficial effects on human health. They should provide a diverse, well-functioning gut microbiota, which is supposed to support optimal recovery of energy from food and its proper storage in the body. It is also supposed to increase the efficiency of the gut barrier, thereby decreasing internal endotoxemia.

## 4. Reducing Symptoms of Lactose Intolerance

Lactose is a disaccharide containing the monosaccharides glucose and galactose. Lactose occurs naturally and almost exclusively in milk from mammals, where it is the main carbohydrate and is often referred to as “milk sugar”. The average lactose content of cow’s milk is about 4.5%. Lactose can be hydrolyzed to glucose and galactose (its constituent monosaccharides) under the action of the enzyme ß-galactosidase (lactase). Lactose intolerance is an abnormal reaction of the human body after consuming products that contain lactose. It can be caused by a deficiency or complete absence of the digestive enzyme lactase, which breaks down lactose into simple sugars: glucose and galactose. If there is not enough of this enzyme in the human body, then non-digested lactose ferments in the large intestine under the influence of bacteria. This, in turn, causes the formation of acids and gases that cause unpleasant digestive discomfort. People who suffer from lactose intolerance experience stomach problems when they drink milk or consume milk products because they do not produce enough lactase amount in their small intestines to digest the milk sugar, which is lactose [73]. Fortunately, many studies have demonstrated that people who do not tolerate lactose can consume fermented milk products like yogurt, kefir, or acidophilic milk. Lactic acid bacteria contained in fermented milk beverages are able to produce lactase, which breaks down lactose in the digestive system. Studies have shown that probiotics such as *Lactobacillus* spp., *B. longum*, and *B. animalis* possess a beneficial influence on people suffering from lactose intolerance when they consume a probiotic yogurt comprising *L. acidophilus* and *Bifidobacterium* spp. [74]. Another study proved that probiotic yogurt supplemented with *L. acidophilus* and *Bifidobacterium* spp. may significantly decrease lactose intolerance symptoms. This means that the consumption of probiotic yogurt may serve as the therapy of choice for lactose intolerance in patients [75]. Low levels of the enzyme lactase cause lactose intolerance, which makes it difficult to digest lactose contained in dairy products. Yogurt has less lactose than non-fermented milk because lactic acid bacteria used as a bacterial starter for yogurt production digests some amount of lactose, so its level in yogurt is lower compared to milk that is not fermented [76].

Symptoms of lactose intolerance occur when the ability to break down lactose to galactose and glucose is reduced, most often due to reduced lactase activity in the small intestine. Depending on the cause, four main types of lactose intolerance can be distinguished: primary, secondary, congenital, and developmental [77]. Secondary lactose intolerance is attributable to a partial, rarely complete decrease in lactase activity in the course of diseases, leading to damage to the small intestinal mucosa or a reduction in its surface area. Congenital lactose intolerance is extremely rare. The underlying cause of the disease is a complete or almost complete lack of the ability to synthesize lactase. Developmental lactose intolerance results from immaturity of the small intestinal mucosa and occurs in premature infants born before 34 weeks of gestation. The most frequently met form of lactose intolerance is primary lactose intolerance (adult-type hypolactasia), which is inherited in an autosomal recessive manner and, in the Caucasian population, is associated with a C/T polymorphism of the lactase gene [78,79]. Both the age at which lactase activity is reduced and the prevalence of lactose intolerance in adulthood depends on ethnicity. It is worth noting that in a certain percentage of people, lactase activity remains relatively high throughout life.

The lactose content of dairy products varies and depends mainly on how they are produced. Dairy products produced by fermentation contain less lactose, which is a substrate for bacteria. As a result of their action, lactose is transformed into lactic acid as well as other substances (e.g., diacetyl, acetic acid, ethanol, carbon dioxide), giving dairy products their characteristic sensory properties. It is important to try to include low-lactose dairy products with a high calcium content in the diet of a person with lactose intolerance. Such products include hard cheese and molded cheese, as well as fermented milk beverages. It is worth noting that the lactose content of hard cheeses is inversely proportional to their ripening time. Lactose tolerance depends on many factors, including how and when dairy products are consumed. A specific portion of lactose consumed at one time may produce significant clinical symptoms while spreading it over several meals, and especially consumed in the company of other products, may lead to leveling or even disappearance of clinical symptoms. The degree of lactose tolerance also depends on the rate of gastric emptying and passage time through the small intestine [80].

It is well known that people with lactose intolerance can consume fermented dairy products better than dairy products that are not fermented with lactic acid bacteria. The concentration of lactose before the fermentation process in the mixture used to prepare yogurt, depending on the additives used, is about 6%. One of the changes that occurs as a result of fermentation is a 20–30% reduction in lactose content. It is broken down by lactase, produced by bacterial microflora. However, the final change in lactose content may not be as significant when powdered milk is used as an additive in yogurt. Other factors are also important. The same amount of lactose contained in yogurt is better tolerated than in milk. Bacteria included in the microflora of yogurt, such as *L. bulgaricus* or *S. thermophilus*, produce lactase, increase the pool of its activity in the intestine, and thus support the digestion of lactose. Products containing live bacterial cultures are therefore recommended. Lactase is relatively resistant to environmental factors. However, heating yogurt significantly reduces its activity. Thus, heat-treated yogurts are less beneficial for people with lactose intolerance. The increased tolerance of lactose consumed with yogurt may also be explained by a direct increase in lactase activity in the small intestine. Animal studies indicate that lactic acid bacteria can induce lactase activity in intestinal epithelial cells. The supply of a lactic-fermented beverage to mice has been shown to stimulate the regeneration of intestinal villi, as well as an increase in lactase activity [81]. The management of probiotics was efficient at improving adult lactose intolerance, and it is expected that the results of this study could help improve the nutritional status of adults by increasing their consumption of milk and dairy products in the future [82]. Lactose-free dairy products could be recommended as alternatives for the alleviation of lactose intolerance and for the promotion of human health and wellness [83]. Another study also demonstrated that the consumption of ice cream containing high or low concentrations of *B. bifidum* 900,791 improves lactose tolerance in hypolactasic subjects [84]. Accumulating evidence has indicated that probiotic bacteria present in milk products can be used to reduce the clinical signs of lactose intolerance [74,85]. Another study highlighted the importance of selected probiotics and vitamin B6 to alleviate symptoms and gut dysbiosis in lactose-intolerant patients with persistent functional gastrointestinal symptoms [86]. However, there are some limitations regarding the wide heterogeneity between the studies examining the effect of probiotics consumption on reducing the symptoms of lactose intolerance [87]. The promising therapeutic influence of probiotic yogurt intake to reduce symptoms of lactose intolerance is presented in Table 2.

## 5. Reducing the Risk of Diabetes

Dairy products, especially fermented milk beverages, constitute a great origin of vitamins, calcium, phosphorus, magnesium, vitamin D, and some fatty acids. It is proved that the consumption of milk products is connected with a decreased occurrence of type 2 diabetes [88]. A special role in the prevention of type 2 diabetes is played by probiotic yogurt intake. Everyday yogurt consumption can also reduce the risk of the incidence of type 2 diabetes and cardiovascular disease [89,90]. It is well known that probiotic bacteria in fermented milk can reduce inflammation or promote insulin effectiveness. Moreover, the consumption of probiotic yogurt may reduce the intestinal flora imbalance and help patients with chronic liver disease experience. Subjects in the tested group consumed three times per day for 2 weeks probiotic yogurt with *B. bifidum*, *L. acidophilus*, *L. delbrueckii* subsp. *bulgaricus*, and *S. thermophilus*. Such treatment of patients successfully reduced liver conditions, particularly in diabetes [91]. The research proved that because of the probiotic yogurt with high nutritional characteristics and low glycemic index, yogurt may help treat diabetes [92].

Insulin resistance and type II diabetes are the most common disorders of carbohydrate metabolism in the world. They are associated with abnormal production of the hormone insulin by the pancreas or lack of tissue sensitivity to it. In Poland, an average of 2 million people have diabetes, but about 25% of sufferers are undiagnosed. According to the Central Statistical Office, 5073 people died of diabetes in 2018 and 10,834 in 2022 [93]. The data show an upward trend in deaths despite the widely used pharmacotherapy for diabetes. Many clinical studies suggest a positive effect of probiotic therapy in slowing the progress of resistance insulin and, thus, type II diabetes [94].

Moreover, scientific reports suggest that probiotic therapy may offset the negative effects of pharmacotherapy, commonly used during type II diabetes. Insulin is a secretory hormone of the β cells of the pancreatic islets (Langerhans). It is composed of proinsulin (insulin precursor), which is composed of amino acid chains A, B, and C. Insulin secretion in the body occurs continuously. Its highest concentration is recorded in the morning hours. Glucose increases insulin production. Rapid insulin secretion from β-cells occurs when blood glucose levels rise suddenly [95].

Reduced sensitivity of tissues to insulin (insulin resistance) results in the inability of glucose isolated by meal consumption to be absorbed into tissues in combination with insulin. Specific glucose transporters (GLUTs), which are located on the surface of peripheral tissues, do not catch the presence of insulin, which impedes the absorption of glucose into cells. This condition leads to hyperglycemia [96]. It results in the relentless production of insulin, causing pancreatic β-cell hypertrophy.

In the long term, this situation can lead to their death, leading to type II diabetes. Proper insulin function is also dependent on hormones. Excessive insulin secretion activates the secretion of factor IGF-1, which is produced under the influence of growth hormone. The activity shown prevents hypoglycemia caused by excessive insulin secretion. This hormone inhibits insulin secretion and synthesis of glycogen in the liver, as well as increases the uptake of circulating glucose in the peripheral blood [97]. Glucagon, glucocorticosteroids, and catecholamines also act synergistically with insulin, inhibiting insulin secretion and glucose synthesis [98]. Insulin resistance is a metabolic disorder in which pathological sensitivity of adipocytes (adipose tissue cells) and cardiomyocytes (cardiac muscle cells) to insulin is observed [97]. It results from impaired working and alarming of insulin receptors, elevated levels of insulin-binding antibodies, or incorrect structure of the insulin molecule. The main factor in the onset of insulin resistance is obesity. It is also found that the risk of this metabolic disorder increases with age [99,100].

Insulin resistance is a condition that premises the onset of type II diabetes. The disorder is characterized by excessive insulin secretion by pancreatic β cells, leading to hyperinsulinemia. This condition is compounded by a diet that is rich in fats and carbohydrates. If pancreatic β-cells are damaged, insulin is no longer produced by them, resulting in the development of a prediabetic state and subsequent type II diabetes. In the course of insulin resistance, as a rule, pharmacotherapy is not used. A patient with this disorder should change his eating habits and lifestyle. Weight reduction, increased physical activity, and the introduction of a low glycemic index diet are recommended. Reducing the energy supply of the diet may be more effective with the help of synbiotic supplementation. They affect the hormones adiponectin and leptin. These hormones are produced by so-called white adipose tissue. Adiponectin acts to increase insulin sensitivity. It also has anti-inflammatory properties that affect weight reduction or minimize the risk of type II diabetes. Leptin reduces hunger and affects insulin secretion in the pancreas. In obesity, which often occurs along with insulin resistance, the action of the aforementioned hormones can be affected [101].

Excessively high levels of leptin in the blood can cause tissue resistance to its action, resulting in excessive appetite and weight gain [102]. Noormohammadi et al. carried out a comprehensive analysis that looked at whether synbiotics affect the levels of the aforementioned hormones. They found that synbiotics can reduce blood leptin levels by decreasing its release by adipocytes, as well as indirect activation of peroxisome proliferator-activated receptor (PPAR), which reduces food intake and leads to weight reduction. This effect reduces tissue sensitivity to insulin. In addition, probiotic bacteria of the genera *Lactobacillus* and *Bifidobacterium* reduce the hydrolysis of leptin in the colon, which increases its concentration. Too high a concentration of leptin affects the activation of oxytocin in the hypothalamus. Activation of oxytocin and its long-term elevated concentration increases leptin resistance, which is very often observed among obese patients. Probiotic bacteria, such as *Lactobacillus reuteri*, have been shown to increase oxytocin production, leading to improved regulation of its homeostasis and affecting weight normalization [101].

Adiponectin has anti-inflammatory, antiatherosclerotic, and anti-diabetic effects. Its elevated levels in the body improve tissue sensitivity to insulin. Kim et al. [103] demonstrated that a strain of *L. rhamnosus* GG administered to mice for 13 weeks increased adiponectin production and activated the adenosine monophosphate-activated protein kinase (AMP-activated protein kinase or AMPK) signaling pathway and thus improved tissue sensitivity to insulin. On the other hand, Raji et al. [104] proved that adiponectin levels in overweight or obese women increased after synbiotic therapy and a low-calorie diet.

An interesting study was conducted at the University of Cambridge. The scientists tested whether a typical Western diet, characterized by high-fat products, while supported by probiotics, can affect the occurrence of syndromes, including resistance to insulin. To this end, the study group consumed a high-fat diet along with a probiotic containing the bacterial species *L. casei*. The control group did not consume the probiotic. The results obtained support the thesis that changing the composition of the intestinal microbiota effectively inhibits the appearance of immunity to insulin in human beings [105].

It was found that despite the consumption of a high-fat diet, fasting blood glucose remained in the typical range in the group of people consuming the probiotics. In addition, probiotic therapy with *L. casei* did not cause cellular resistance to insulin despite the subjects’ consumption of about 270 g of fat per day. Conclusions were drawn that supporting a typically Western diet with probiotics may reduce the possibility of insulin resistance. However, such an intervention should not replace changes in dietary habits in overweight or obese individuals, and probiotic therapy may be a supportive element in changing dietary habits [105].

Type II diabetes is a consequence of insulin resistance. It is characterized by hyperglycemia that results from a defect in insulin secretion. The pathogenesis of type II diabetes centers on insufficient insulin production by pancreatic β cells. The disease most often affects people over the age of 40 and is more common in overweight individuals than in normal-weight individuals [94,106]. Risk factors that influence the development of type II diabetes, in addition to obesity and overweight, include genetic predisposition, low physical activity, hypertension, elevated HDL cholesterol and triglycerides, and in women, a history of gestational diabetes and polycystic ovary syndrome. Individuals diagnosed with fasting glucose levels indicative of pre-diabetes should be introduced to appropriate physical exercises together with dietary treatment to affect weight reduction. Scientific reports suggest the effect of probiotics on reducing the risk of type II diabetes. Zhang et al. tested the effect of *L. casei* bacteria on the influx of chloride ions to reduce susceptibility to diabetes. The mouse study confirmed that high chloride ion concentrations in the pancreas are necessary for β-cell cell membrane activity and insulin release from the cells [107].

However, some studies have revealed that probiotic consumption has not contributed to a significant health effect in the treatment of diabetes. The study assessed the effectiveness of probiotics on glycemic control in type 2 diabetes patients. 16-week probiotic supplementation (25 mL of beverage containing over 10^8^ CFU/mL of *Lactobacillus*, four times per day) resulted in no profitable effects on glycemic control, profile of lipids, as well as weight [108].

The examples of therapeutic effects of probiotics intake in reducing the risk of diabetes are presented in Table 3.

The effect of *L. casei* bacteria causes the release of lipopolysaccharides into the blood through a mechanism that regulates chloride production [107]. Kupffer cells, located in the liver, are composed of a chloride channel coupled to glycine. Glycine is an amino acid classified as a glycine receptor agonist. Their activation affects, among other things, Kupffer cells by reducing their sensitivity to pro-inflammatory factors (TNF-alpha). Ingestion of *L. casei* bacteria in the form of a probiotic causes an influx of chloride ions, which have an inhibitory effect on the development of type II diabetes. The authors focus attention on the fact that the loss of chloride ions in tissues, which affects the pathogenesis of type II diabetes, may also be related to comorbidities in the course of diabetes, such as insulin sensitivity, myotonia, and kidney stones [107]. Overweight and obesity directly affect the risk of insulin resistance. In turn, insulin resistance is the first step toward the development of type II diabetes. Proper prevention and use of probiotics may lead to health benefits.

The risk of insulin resistance among obese patients who consume a typical high-fat diet can be minimized by probiotic therapy with *L. casei*. These bacteria have a protective effect on the onset of cellular resistance to insulin. Increased appetite can be influenced by concentrations of leptin, the satiety hormone, that are too high. In turn, concentrations that are too high influence the increase in food consumption, leading to obesity and, consequently, immunity to insulin. In patients already diagnosed, it is worthwhile to use probiotic therapy with *Lactobacillus* and *Bifidobacterium*, which reduce the hydrolysis of leptin in the colon, which lowers the leptin concentration. In addition, the *L. rhamnosus* GG strain increases adiponectin levels, which can improve tissue sensitivity to insulin.

In patients at risk of developing type II diabetes, it is recommended to include probiotic therapy with *L. casei*. The consumption of metformin affects the gastrointestinal tract by decreasing the amount of these bacteria. In turn, pharmacotherapy with drugs from the α-glucosidase inhibitor group can cause a decrease in the number of bacteria from the *Firmicutes* cluster. The disturbed ratio causes dysbiosis manifested by diarrhea. In patients treated with α-glucosidase inhibitors, it is worth introducing probiotic therapy recommended for diarrhea. These strains include *L. rhamnosus* GG and *S. boulardii* [107]. Diabetes cannot be completely cured. However, there are various methods to control diabetes. A promising strategy is the use of probiotics for diabetes intervention [109]. As a potential new intervention target for the treatment of diabetes, probiotics may participate in the regulation of energy metabolism through various mechanisms, namely, reducing chronic low-grade inflammation, regulating intestinal flora, reducing oxidative stress, increasing bacterial bioactive peptides and improving insulin resistance, to achieve the purpose of regulating blood glucose. Probiotics are considered economical and safe alternatives to treat chronic diseases and improve human health [110]. The administration of *L. paracasei* HII01 was shown to be effective in the improvement of metabolic variables in diabetes [111]. Another study showed that *L. paracasei* IMC 502 significantly decreased blood glucose and lipid levels, improved insulin resistance and glucose intolerance, and repaired pancreatic and hepatic tissue damage. This probiotic conferred beneficial outcomes in the gut microbiome of diabetic mice, which induced the transformation of short-chain fatty acids (SCFAs), further enhanced the secretion of downstream hormones, and ultimately ameliorated the inflammatory response [112]. Probiotics significantly improved blood glucose and blood lipid parameters, as well as the morphological changes of the pancreas, liver, and kidney in diabetic mice [113]. One of the important incretins in the improvement of diabetes is glucagon-like peptide (GLP-1), which is secreted by the gut and reduces the apoptosis of pancreatic β-cells and improves insulin sensitivity. The study showed that probiotics and resveratrol significantly decreased (*p* < 0.001) glucose and insulin resistance and increased (*p* < 0.001) GLP1 and total antioxidant capacity compared to the diabetic group. Treatment with probiotics and resveratrol also returned intestinal histological changes in diabetic rats to normal [116]. The consumption of probiotics could be a useful intervention to improve cardiometabolic outcomes through reduced inflammation and oxidative stress in patients with prediabetes and type 2 diabetes [117,118,119]. The study showed that *L. plantarum* HAC01 was efficient in treating hyperglycemia and type 2 diabetes mellitus in mice with high-fat diets and streptozotocin-induced diabetes [114]. *L. plantarum* Y15 administration to type 2 diabetes mice could improve the biochemical indexes related to diabetes and reduce the production of pro-inflammatory cytokines. *L. plantarum* Y15 administration could reshape the structure of gut microbiota. *L. plantarum* Y15 administration could also regulate the expression levels of the inflammation and insulin signaling pathway-related genes [115]. The results of many studies suggested that probiotics may serve as potential agents for ameliorating type 2 diabetes [120,121,122,123,124,125,126].

## 6. Inhibition of the Growth of Intestinal Pathogens

Probiotics are living microorganisms that constitute the natural human intestinal microbiota and ensure its homeostasis. They have the ability to adhere to the intestinal epithelium, colonize the intestine, and antagonize the action of typical pathogens of the gastrointestinal tract. At the same time, they do not exhibit toxic and pathogenic effects on the host organism. Probiotic properties are possessed by strains of lactic acid bacteria, such as *Bifidobacterium*, *Lactobacillus*, *Enterococcus*, *Propionibacterium*, *Lactococcus*, *Saccharomyces*, *Bifidus* or *Streptococcus salivarius*, and the yeast *S. boulardii*. In the gastrointestinal tract, they have the ability to inhibit the growth of pathogenic bacteria through the production of organic acids and hydrogen peroxide and by a competitive mechanism of microbial adhesion to the intestinal epithelium. Organic acids formed in the gastrointestinal tract during the metabolism of probiotics, such as pyroglutamic acid, inhibit the growth of Gram-negative bacteria, such as *Pseudomonas* and *Enterobacter*. In turn, hydroxyl radicals produced by *Lactobacillus lactis* inhibit the growth of *Staphylococcus aureus*. In addition, the acidification of intestinal contents by probiotics inhibits the growth of certain pathogenic bacteria.

In the lumen of the digestive tract, probiotics compete with other microorganisms for nutrients; probiotics consuming simple sugars inhibit the growth of *C. difficile*. In the digestive tract, they also increase the secretion of mucins, which are glycoproteins that have a protective effect in intestinal infections. Such an effect has been proven for the *L. acidophilus*. In the gastrointestinal tract, probiotics are characterized by a very wide range of actions. At the cellular level, their involvement in increasing the phagocytic activity of macrophages and lymphocytes and activating natural cytotoxic cells is highlighted [127].

Numerous clinical trials are currently being conducted to determine the beneficial effects of probiotics in conditions such as bacterial infectious diarrhea, viral infectious diarrhea, post-antibiotic diarrhea, and “traveler’s” diarrhea. Acute infectious diarrhea causes about 5% of hospitalizations of children in Poland. Their cause is most often rotavirus or adenovirus, less often bacteria such as *Salmonella*, *Shigella*, *Yersinia*, *E. coli*, or parasites such as *Giardia* [128,129]. There have been a number of studies that show that the supply of certain probiotics can shorten the duration of diarrhea. Isolauri et al. [130] showed that *Lactobacillus* GG significantly reduces the duration of rotavirus diarrhea, while Majama et al. [131] compared the effectiveness of their treatment with different types of lactic acid bacteria. The duration of diarrhea in patients receiving *Lactobacillus* GG was 1.8 days, significantly shorter than those receiving *L. rhamnosus* (2.8 days) and *S. thermophilus* (2.6 days). Another probiotic showing a beneficial effect on the duration of diarrhea in children was *L. reuteri* [132].

Saavedra et al. [133], on the other hand, showed the beneficial effects of *B. bifidum* and *S. thermophilus* in preventing rotavirus diarrhea in infants. Their incidence was significantly lower in infants receiving probiotics (7%) than in the placebo group (35%). Diarrhea associated with antibiotic use occurs in about 11–40% of pediatric patients [134] and depends on the type of antibiotic used (the highest risk is with beta-lactam antibiotics). The cause of post-antibiotic diarrhea is the regrouping of the intestinal microbiota and the compounds it synthesizes. As a result, there is an excessive accumulation of undesirable microorganisms, such as *C. difficile*, *Clostridium perfringens*, and *Staphylococcus aureus* [129]. Avrola et al. [135] demonstrated that the incidence of post-antibiotic diarrhea diminished twofold in children receiving a preparation containing *Lactobacillus* GG (*n* = 61) compared to the group receiving placebo (*n* = 58). Vanderhoof et al. [136] described an even greater protective potential of probiotics. Incidents of diarrhea in the children studied, thanks to the supply of *Lactobacillus* GG, occurred more than three times less often in comparison to the control group (*n* = 188). In contrast, Piątkowska et al. [137] showed that supplementation with *Saccharomyces boulardi* reduced the risk of diarrhea in children by more than 10%. Similar results regarding the use of *Saccharomyces boulardi* in children were obtained by Kotowska et al. [138]. Szamański et al. [139] proved that the supply of *B. longum*, *L. rhamnosus*, and *L. plantarum* to patients aged 5 months to 16 years has an effect on reducing the number of stools per day.

The modulation of the gut microbiota through reshaping host–microbiota interactions can be achieved with strategies such as probiotics and personalized nutrition as adjunctive therapy. Pathogenic microorganisms alter the homeostasis of the gut microbiota, leading to an increased risk of related diseases. Probiotics inhibit these pathogens by stimulating epithelial barrier function, secreting anti-microbial components, competitively excluding pathogens by binding sites, and limiting their access to nutrients [140]. Many studies implied that a small number of genetic mutations or even a single-nucleotide variant in the microbial genome could significantly alter the pathogenic behavior of gut bacteria and affect host health. The study demonstrated that probiotic consumption can lead to widespread single-nucleotide variants in the native microbiota. The study substantially extended the understanding of the co-evolution of the probiotics and the indigenous gut microbiota, highlighting the importance of assessment of probiotics efficacy and safety in an integrated manner [141]. The study showed that probiotics can interact with pathogens in foods and in the hosts. Probiotics use different mechanisms to control enteric pathogen colonization. They are able to produce organic acids, bacteriocins, and hydrogen peroxide, which have an inhibitory effect on the growth of many pathogens in the gut [142]. The promising therapeutic effects of probiotic yogurt consumption in the inhibition of the growth of intestinal pathogens are presented in Table 4.

## 7. Protection Against Cancer

The composition of the gut microflora remains dependent on various factors, such as age, dietary habits, physical activity levels, drug treatment, and surgical procedures. The intestinal microbiota contributes to the process of cancer formation. Intestinal dysbiosis, or microflora imbalance, is otherwise known as qualitative and quantitative changes in the intestinal microbiota, which are observed in cancer. Increased amounts of *Fusobacterium nucleatum*, *Bacteroides fragilis*, *Enterococcus faecalis*, *Streptococcus bovis*, and *Peptostreptococcus anaerobius* have been reported in colon cancer patients. Studies show that the abundance of several specific bacteria, such as *Lactobacillus*, *Escherichia*, *Shigella*, *Nitrospirae*, *Burkholderia fungorum*, and *Lachnospiraceae*, is increased in patients with gastric cancer.

The main bacterial clusters residing in the human gastrointestinal tract are *Firmicutes* (64%), *Bacteroidetes* (23%), *Proteobacteria* (8%), and *Actinobacteria* (3%) [143]. The environment of the gastrointestinal tract and its conditions are variable, so the type and density of individual microorganisms depend on the location [144]. The composition of the microbiota varies depending on the gastrointestinal tract section. The oral cavity is dominated by bacteria, including *Streptococcus*, *Peptococcus*, *Staphylococcus*, *Bifidobacterium*, *Lactobacillus*, and *Fusobacterium* [145]. The stomach has an acidic pH, which means that there can be few microorganisms capable of surviving in such an environment, such as *Helicobacter pylori* and *L. plantarum* 299v. The small intestine is dominated by Gram-positive and Gram-negative anaerobic bacteria. In contrast, the large intestine is a place where numerous bacteria can thrive, and Gram-negative aerobic bacteria predominate [144].

Intestinal dysbiosis is defined as a disorder in the quantitative and qualitative composition of the gastrointestinal microbiota [144]. These disorders can contribute to a number of diseases, including inflammatory bowel disease (Crohn’s disease, ulcerative colitis), chronic kidney disease, and gastrointestinal cancers. The carcinogenic activity of the intestinal microbiota is associated with negative metabolic transformations induced by intestinal microorganisms. As a result of these transformations, toxic, mutagenic, and carcinogenic substances can be formed [145]. The gastric microbiota is mainly composed of bacteria: *Firmicutes*, *Bacteroidetes*, *Fusobacteria*, *Actinobacteria*, and *Proteobacteria* [146]. Increased amounts of *Lactobacillus*, *Escherichia*, *Shigella*, *Nitrospirae*, *Burkholderia fungorum*, and *Lachnospiraceae* are observed in patients with gastric cancer [145,146].

The pathogenesis of colorectal cancer also includes disturbances in the composition and activity of the intestinal microbiota. Bacteria involved in the carcinogenesis of colorectal cancer are *Fusobacterium nucleatum*, *B. fragilis*, *Escherichia coli*, *Enterococcus faecalis*, *S. bovis*, and *Pept. anaerobius*. The effect of individual bacteria as carcinogens varies. For example, a major part of the enterotoxigenic pathogenicity of *B. fragilis* (ETBF) is the synthesis of *B. fragilis* toxin (BFT) [147]. This toxin activates signal transducer and activator of transcription 3 (STAT3) proteins. STAT proteins are a family of transcription factors involved in the transduction of extracellular signals and the direct regulation of transcription [147,148]. The function of these proteins is to control the expression of genes involved in cell survival, proliferation, chemoresistance, and angiogenesis. STAT3 protein is a latent cytoplasmic transcription factor activated by cytokines and growth factors, among others. STAT3 protein is involved in the generation of cells responsible for inflammation: Th17. Th17 cells produce IL-17 and activate the transcription factor NF-kB and the Wnt signaling pathway. Abnormalities in the Wnt pathway are observed in many diseases, including but not limited to cancer [149].

Probiotic strains have anti-cancer properties by, among other things, binding carcinogens, inhibiting the activity of bacterial nitroreductases, lowering the concentration of secondary bile acids, and inhibiting the synthesis of fecal enzymes (e.g., p-glucuronidase) [150]. The gastrointestinal microbiota can be modified not only with probiotics but also with prebiotics and synbiotics [151]. Prebiotics stimulate the growth or activity of one or a specific number of bacterial genera in the colon and benefit the health of the host [152,153]. Prebiotics include inulin, lactulose, and fructooligosaccharides, among others. It should be noted that prebiotics provide a medium for probiotics and stimulate their growth, but unlike them, they do not have microorganisms in their composition [152]. Synbiotics exert a beneficial effect on the gastrointestinal microbiota by stimulating probiotics with prebiotics and impeding the proliferation of pathogenic bacteria. Synbiotics inactivate nitrosamines and carcinogens [152].

Today, colorectal cancer is the third most common cancer worldwide. Etiopathogenesis includes genetic and environmental factors, such as a poor diet (with high saturated fatty acids, consumption of red meat, and low dietary fiber intake) and a sedentary lifestyle [154]. Most colorectal cancers are sporadic and not genetically determined. Familial polyposis syndrome and Lynch syndrome (hereditary colorectal cancer unrelated to polyposis) account for 5% of all cases. Genetic disorders, usually inherited in an autosomal dominant manner, contribute to the growth of such kinds of cancer [154,155].

Counteracting colorectal cancer includes chemoprevention and screening, so-called screening tests [154]. Vitamins may have a potential chemopreventive effect. So far, regular consumption of acetylsalicylic acid has been shown to reduce the risk of developing adenomas and colorectal cancer. However, acetylsalicylic acid increases the incidence of gastrointestinal bleeding and hemorrhagic stroke [155]. Chemopreventive factors also include a diet with little red meat and plenty of vegetables, fruits, and dietary fiber. Probiotic strains can also be used in the control of colorectal cancer [154]. In the in vitro studies, probiotics have been observed to exhibit a number of anti-cancer properties, including inactivation of mutagens and carcinogens, lowering of intestinal pH, inhibition of tyrosine kinase pathway activity, immunomodulation and modification of the gastrointestinal microbiota [156].

In an animal model study, *Ligilactobacillus salivarius* strain UCC118 was shown to reduce levels of enterococci and *C. perfringens* [157]. It should be noted that *C. perfringens* does not infect healthy cells but produces toxins and extracellular enzymes responsible for pathogenicity. To date, 20 *C. perfringens* toxins have been described, which can be divided into four main categories: cell membrane-damaging, pore-forming, intracellular toxins, and hydrolytic enzymes. Strains of *C. perfringens* are divided into five subtypes: A-E. However, subtype A is the most common cause of food poisoning [158]. On the other hand, a study by Horie et al. [159] reported that a mixture of pro-biotic strains (*Streptococcus faecalis*, *Clostridium butyricum*, *Bacillus mesentericus*) reduced the formation of DNA adducts in the colonic epithelium.

The *L. plantarum* strain reduces the enzymatic activity of different bacteria and the concentration of bile acids in feces. *L. rhamnosus* 231 reduces the activity of fecal nitroreductase, nitroreductase, and glutathione S-transferase. It also causes an increase in glutathione reductase activity. *L. acidophilus* KFRI342 reduces fecal *Escherichia coli*, glucuronidase, and glucosidase, as well as triglyceride levels [160]. In vitro studies have also confirmed the antinociceptive effects of synbiotics and prebiotics. A synbiotic containing *Bifidobacterium lactis* and resistant starch exerts a pro-apoptotic effect in response to a carcinogen. In vivo studies also confirm the antitumor activity of probiotics [156]. The probiotic strain *S. boulardii* prevents epidermal growth factor (EGF)-induced proliferation and enhances apoptosis in vitro and in vivo. In contrast, the *Bifidobacterium polyfermenticus* strain inhibits ErbB2 and ErbB3 activity [161]. ErbB2 is a trans-membrane receptor tyrosine kinase encoded by a gene located in chromosomal region 17q12. Overexpression of ErbB2 and ErbB3 plays an important role in oncogenesis [162].

Clinical studies with patients have shown that probiotics administered in the preoperative period, as well as during chemotherapy, reduced treatment side effects and modified the gastrointestinal microbiota. In a study by Hattak et al. [163], it was observed that the administration of a probiotic mixture (*L. rhamnosus* LC705, *Propionibacterium freudenreichii* ssp. *shermanii* JS) reduced glucosidase activity and modified the gastrointestinal microbiota. In an animal model study, Jacouton et al. [164] showed that *L. casei* strain BL23 prevents the development of colorectal cancer, as it has immunomodulatory properties (reduces anti-inflammatory interleukin-IL-22) and has antiproliferative effect by regulating caspases (7 and 9) and the pro-apoptotic protein Bik.

The other study highlights the pivotal role of probiotics in preventing and alleviating colon cancer. Probiotics are able to use the diverse mechanisms through which they contribute to mitigating colon cancer, including inhibiting cell proliferation and promoting apoptosis, enhancing the immune response, regulating the intestinal microbiota and their metabolic processes, strengthening the intestinal barrier, and boosting antioxidant effects. Probiotics themselves exhibit anticancer activity, and the metabolites they produce, such as bacteriocins and short-chain fatty acids, possess the capacity to inhibit colon cancer [165]. Probiotics have shown promising anticancer effects, which, combined with their ability to fight the side effects of synthetic drugs, may provide potentially useful treatments in the future. The studies at the cellular level revealed the occurrence of apoptosis under the effect of the probiotic mix. Collectively, the experimental data show that probiotics have the ability to efficiently fight cancer proliferation. Further studies should reveal their efficiency in vivo and eventually in clinical settings [166]. Daily consumption of the probiotic *L. casei* strain was shown to impair tumor growth. This effect could be attributed to the production of Th1 immunostimulatory cytokines and secretion of chemoattractant molecules in tumor tissue, accompanied by a migration of cytotoxic T cells in the tumor [167]. Treatment with *L. coryniformis* MXJ32 could significantly inhibit the total number of tumors and the average tumor diameter [168]. The effects of the probiotic mixture on proliferation and metastasis of mouse colon cancer CT26 cells were assessed by probiotics and cells co-culture assay, Cell Counting Kit-8 assay, colony formation assay, wound-healing assay, as well as migration and invasion assays. The probiotic mixture significantly inhibited the proliferation, invasion, and migration ability of CT26 cells compared to the control cells [169]. The promising therapeutic effects of probiotics administration in the protection against cancer are presented in Table 5.

However, some studies have revealed that probiotics consumption has not contributed to a significant health effect in the treatment of cancer. Although probiotics have shown success in preventing the development of experimental colitis-associated colorectal cancer (CRC), the beneficial effects of interventional treatment are relatively unknown. The study showed that interventional treatment with VSL#3 probiotics alters the luminal and mucosally adherent microbiota but does not protect against inflammation or tumorigenesis in mouse models [170]. Evidence from in vitro and animal studies supports the potential chemopreventive properties of prebiotics and probiotics. However, human studies using pro and prebiotics have not provided consistent results in support of their efficacy in preventing cancer. More randomized controlled human trials need to be conducted with a wide range of populations with stringent controls. These control measures are difficult because genetics and immune response potential are varied throughout the population [171].

**Table 5:** Promising therapeutic effects of probiotics administration in the protection against cancer.

Subject	Effect	References
Human	Probiotic strains have anti-cancer properties by, among other things, binding carcinogens, inhibiting the activity of bacterial nitroreductases, lowering the concentration of secondary bile acids, and inhibiting the synthesis of fecal enzymes (e.g., p-glucuronidase).	[150]
Human	*B. lactis* and resistant starch exert a pro-apoptotic effect in response to a carcinogen. In vivo studies also confirm the antitumor activity of probiotics.	[156]
Human	Administration of a probiotic mixture (*L. rhamnosus* LC705, *Propionibacterium freudenreichii* sp. *shermanii* JS) reduced glucosidase activity and modified the gastrointestinal microbiota.	[163]
Mice	In an animal model study, Jacouton et al. showed that *L. casei* strain BL23 prevents the development of colorectal cancer, as it has immunomodulatory properties (reduces anti-inflammatory interleukin-IL-22) and has an antiproliferative effect by regulating caspases (7 and 9) and the pro-apoptotic protein Bik.	[164]
Human	The other study highlights the pivotal role of probiotics in preventing and alleviating colon cancer. Probiotics themselves exhibit anticancer activity, and the metabolites they produce, such as bacteriocins and short-chain fatty acids, possess the capacity to inhibit colon cancer.	[165]
In vitro study	Probiotics have shown promising anticancer effects, which, combined with their ability to fight the side effects of synthetic drugs, may provide potentially useful treatments in the future. The studies at the cellular level revealed the occurrence of apoptosis under the effect of the probiotic mix.	[166]
Mice	Daily consumption of the probiotic *L. casei* strain was shown to impair tumor growth. This effect could be attributed to the production of Th1 immunostimulatory cytokines and secretion of chemoattractant molecules in tumor tissue, accompanied by a migration of cytotoxic T cells in the tumor. However, due to the limitations of pre-clinical mouse models, caution should be taken when interpreting these findings for clinical application; further work in that direction is needed.	[167]
Mice	Treatment with *L. coryniformis* MXJ32 could significantly inhibit the total number of tumors and the average tumor diameter. This probiotic administration prevented the damage to intestinal barrier function by enhancing the expression of tight junction proteins (Occludin, Claudin-1, and ZO-1) and recovering the loss of goblet cells.	[168]
Mice	Colorectal cancer (CRC) is one of the most common cancers, and supplementation of probiotics may be a promising intervention method. *Lactiplantibacillus plantarum* KX041 could significantly inhibit inflammation and tumor formation and induce tumor cell apoptosis.	[172]
Mice	The study showed that interventional treatment with VSL#3 probiotics alters the luminal and mucosally adherent microbiota but does not protect against inflammation or tumorigenesis in mouse models.	[170]
In vitro study	Evidence from in vitro and animal studies supports the potential chemopreventive properties of prebiotics and probiotics. However, human studies using pro and prebiotics have not provided consistent results in support of their efficacy in preventing cancer.	[171]

Probiotics have applications not only at the stage of cancer prevention but also can significantly improve the results of standard anticancer treatment. In addition, they reduce the side effects of enteral feeding, and with long-term probiotic therapy, they can potentially improve the nutritional status of the patient. Probiotic strains also have an immunomodulatory effect, influencing the immune system, and in patients in the postoperative period, they reduce the risk of infection.

## 8. The Efficacy of Probiotic Therapy in the Eradication of *Helicobacter pylori* Infection

Probiotic strains have also been tried for the treatment of peptic ulcer disease, in whose pathogenesis the role of *H. pylori* is well known and documented. Indeed, more and more effective treatment regimens are still being sought. Currently, the most commonly used is a three-drug therapy consisting of a proton pump inhibitor and two antibiotics [129,137,173]. Numerous studies have been conducted to test the efficacy of probiotics in both inhibiting the growth of *H. pylori* and reducing side effects associated with eradication treatment (Table 6). However, the results of clinical trials evaluating the efficacy of probiotics in *H. pylori* eradication are inconclusive. However, the role of probiotics in reducing the incidence of side effects associated with the treatment of the infection is well documented [174]. In a study conducted by Czerwionka-Szaflarska et al. [173], it was proven that the supply of probiotics during *H. pylori* eradication therapy statistically reduces the number of symptoms, such as abdominal pain, nausea, and vomiting, compared to the group not receiving the probiotic (*n* = 60). A study by Sykora et al. [175] using a placebo confirmed the efficacy of *L. casei* in reducing the incidence of side effects of eradication therapy (excluding loose stools) in study patients (*n* = 86). Nista et al. [176] showed that a probiotic preparation containing *Bacillus clausii* significantly reduced the frequency and severity of nausea, abdominal pain, and loose stools as adverse impacts of *H. pylori* removal treatment in patients (*n* = 120).

The efficacy of probiotic therapy in the eradication treatment of *H. pylori* infection has also been confirmed, particularly emphasizing the improved tolerability of treatment due to the lower risk of side effects of the combined antibiotic combination. However, specific probiotic strains should only be selected on the basis of demonstrated clinical efficacy. The best-documented effect has been shown for *S. boulardii* [177]. Modification of the microbiota through the use of probiotics is also a therapeutic pathway in functional diseases, especially irritable bowel syndrome (IBS). In patients with this syndrome, there was a reduction in the severity of symptoms, bloating sensation, and frequency of intestinal gas evacuation as a result of probiotic therapy [178]. The authors suggest the need to administer probiotics in patients with IBS not only “more than 7 days” but at least 4 weeks [179].

However, the most important species responsible for the formation of gastric cancer is *H. pylori*. Gastric cancer is the fifth most common cancer worldwide. The incidence of this cancer is declining, but it remains a relatively common cause of death in Poland, the United States, and Japan [174]. The first-order carcinogen is the bacterium described above, *H. pylori*. There are many different classifications of gastric cancer. Clinically, gastric cancer can be divided into early gastric cancer (EGC) and advanced gastric cancer (AGC). However, the most useful histological division includes two types: Lauren 1 and Lauren 2. It is worth noting that the histological type of cancer is important for the patient’s prognosis [180].

*H. pylori* causes inflammation of the mucosa, mucosal atrophy, and, consequently, the development of gastric cancer. There are several therapeutic methods that can be successfully used to change the composition and activity of the intestinal microbiota. One of them is the administration of probiotic strains, prebiotics, and synbiotics. Probiotics can be administered to prevent the development of gastric and colorectal cancer, which has been shown in many in vivo and in vitro studies. Probiotics reduce the incidence of diarrhea associated with enteral feeding. Probiotic bacterial strains can be included as adjunctive therapy in the treatment of *H. pylori* infection. Probiotics reduce the incidence of postoperative infections, abdominal pain, and radiation diarrhea. This means that probiotics can be used to prevent the development of cancer and can significantly improve the effectiveness of standard cancer therapy [181].

However, *H. pylori* (a Gram-negative, microaerophilic, spiral- or sigmoid-shaped bacillus) is the main pathogenic bacterium responsible for the formation of gastric cancer [176]. Its length is about 2.5–5 pm, and its width is about 0.5–1 pm. It shows the ability to move due to the presence of cilia arranged in a polar pattern. Approximately 70% of people from developing countries and about 30% of people in developed countries are infected with this bacterium [182]. *H. pylori* is one of the main contributors to gastritis and duodenal ulcers [183].

In counteracting gastric cancer, *Helicobacter pylori* eradication is particularly important. A study by Namkin et al. [184] evaluated the efficacy of *S. boulardii* CNCM I-745 in the eradication of *H. pylori* in children. The subjects (*n* = 28) were divided into two groups; the first received the probiotic at a dose of 250 mg/day for one month, and the control group received a placebo. There was no statistically significant difference in achieving eradication between the study groups. Another study observed that oral supplementation of *S. boulardii* CNCM I-745 at a dose of 250 mg, two times/day for 2 weeks, reduced the incidence of diarrhea, an adverse influence of standard removal treatment [185]. Similar results were obtained in a systematic review and meta-analysis by Szajewska et al. [186]. Probiotic strains *L. reuteri* and *L. gasseri*, in combination with proton pump inhibitors, increase the eradication speed of *H. pylori* [186]. The results of a meta-analysis by Tong et al. [187] involving 14 studies also confirmed that probiotics are effective in increasing this rate. Previous studies show that probiotic strains cannot be used as the sole eradication agent, but they are an adjunctive therapy during standard treatment and reduce the side effects of antibiotic therapy [188].

**Table 6:** Promising therapeutic effects of probiotics in the eradication of *H. pylori* infection.

No.	Effect	References
1	The supply of probiotics during *H. pylori* eradication therapy statistically reduces the number of symptoms, such as abdominal pain, nausea, and vomiting, compared to the group not receiving the probiotic.	[173]
2	The probiotic preparation containing *Bacillus clausii* significantly reduced the frequency and severity of nausea, abdominal pain, and loose stools, which are side effects of *H. pylori* eradication therapy in patients.	[176]
3	Probiotic bacterial strains can be included as adjunctive therapy in the treatment of *H. pylori* infection. Probiotics reduce the incidence of postoperative infections, abdominal pain, and radiation diarrhea. This means that probiotics can be used to prevent the development of cancer and can significantly improve the effectiveness of standard cancer therapy.	[186]
4	Probiotic strains *L. reuteri* and *L. gasseri*, in combination with proton pump inhibitors, increase the eradication rate of *H. pylori*.	[187]

## 9. Conclusions

Fermented milk beverages are a very important food category in the structure of the Polish diet. They have health-promoting effects on the human body. The scientific studies cited in this article confirm the health-promoting benefits associated with the consumption of fermented milk beverages. The presence of lactic acid bacteria, including probiotic bacteria in yogurts and kefirs, supports the maintenance of immunity, reduces the incidence of obesity and diabetes, and serves as a weapon in the prevention of cancer. The consumption of fermented milk beverages containing probiotic bacteria, therefore, contributes to the overall improvement of human health and is an essential point in preventing the occurrence of civilization diseases. Probiotic bacteria regulate the immune system, maintain the integrity of the intestinal epithelium, protect the host body from pathogens, and produce a number of metabolites that possess a beneficial influence on human health. Fermented milk drinks are one of the richest sources of probiotic bacteria, which is why it is so important to consume them daily, especially in the context of preventing the development of cancer diseases.

## Figures and Tables

**Table 2:** Promising therapeutic effects of probiotic yogurt consumption in decreasing signs of lactose intolerance.

Subject	Effect	References
Human	The studies have shown that probiotics such as *Lactobacillus* spp., *Bifidobacterium longum*, and *Bifidobacterium animalis* possessed profitable influence on people suffering from lactose intolerance when they consumed a yoghurt including *L. acidophilus* and *B. bifidum*.	[74]
Human	The study proved that yogurt containing *L. acidophilus* and *Bifidobacterium* spp. is capable of significantly decreasing lactose intolerance symptoms. This means that the consumption of probiotic yogurt may serve as the treatment for lactose intolerance in patients.	[75]
Human	Bacteria included in the microflora of yogurt, such as *L. bulgaricus* or *S. thermophilus*, produce lactase, increase the pool of its activity in the intestine, and thus support the digestion of lactose.	[80]
Mice	The supply of a lactic-fermented beverage to mice has been shown to stimulate the regeneration of intestinal villi, as well as an increase in lactase activity.	[81]
Human	The study showed that probiotic management mitigated the symptoms of lactose intolerance.	[82]
Human	Probiotics and fermented milk products influence the metabolic work of colonic microbiota and may diminish the symptoms of lactose intolerance.	[83]
Human	This study demonstrated that the consumption of ice creams with the application of *B. bifidum* 900,791 at high and low levels has improved lactose tolerance in hypolactasic persons.	[84]
Human	The wide heterogeneity between the research studies is shown in this review. One research study inspects the profits of probiotic and prebiotic supplementation, which is why additional clinical trials should be carried out in order to gather more evidence.	[87]

**Table 3:** Promising therapeutic effects of probiotics consumption in decreasing the occurrence of diabetes.

Subject	Effect	References
Human	The intervention group of 40 subjects consumed probiotic yogurt with *B. bifidum*, *L. acidophilus*, *L. delbrueckii* subsp. *bulgaricus*, and *S. thermophilus* three times per day for 14 days. Such treatment of patients successfully lowered liver symptoms, particularly in diabetes.	[91]
Human	The research proved that because of the probiotic yogurt’s high nutritional content and low glycemic index, yogurt may help treat diabetes.	[92]
Human	Many clinical studies suggest a positive effect of probiotic therapy in slowing the progress of insulin resistance and, thus, type II diabetes.	[94]
Human	Probiotic bacteria, such as *L. reuteri*, have been shown to increase oxytocin production, leading to improved regulation of its homeostasis and affecting weight normalization.	[101]
Mice	*L. rhamnosus* GG administered to mice for 13 weeks increased adiponectin production and activated the AMP-activated protein kinase (AMPK) signaling pathway, thus improving tissue sensitivity to insulin.	[103]
Human	Adiponectin amount in overweight or obese women studied rose after synbiotic therapy and a low-calorie diet.	[104]
Human	To this end, the study group consumed a high-fat diet along with a probiotic containing the bacterial species *L. casei*. The control group did not consume the probiotic. The results obtained support the thesis that changing the composition of the intestinal microbiota effectively inhibits the progress of insulin resistance in humans.	[105]
Human	Ingestion of *L. casei* bacteria in the form of a probiotic causes an influx of chloride ions, which possess an inhibitory influence on the occurrence of type II diabetes.	[107]
Human	*Lb. rhamnosus*, *Lactobacillus paracasei*, *Lb. plantarum* and *Bifidobacterium* species were mentioned in many clinical studies providing facts about their anti-diabetic influence. However, anti-diabetic supplements are at the stage of clinical trials, so there is no such product on the market at the moment.	[109]
Human	The usage of hypoglycemic drugs has some limitations and side effects, while probiotics are recognized as safe, economical, and effective. Probiotics, through their good application prospects in the remodeling of intestinal microecological health, are becoming a hot topic of diabetes prevention and treatment.	[110]
Human	The study showed that *L. paracasei* HII01 has a role as an adjuvant treatment in type 2 diabetes by improving hyperglycemia and inflammatory markers. *L. paracasei* positively influences gut microbiota and subsequently improves leaky gut and endotoxemia.	[111]
Mice	The study showed that *L. paracasei* IMC 502 transforms short-chain fatty acids and next increases the secretion of downstream hormones with beneficial outcomes in the gut microbiome.	[112]
Mice	Fourteen probiotic bacteria were identified from fermented camel milk. Blood glucose and lipid parameters were significantly improved, as well as the morphological changes of the pancreas, liver, and kidney.	[113]
Mice	*L. plantarum* HAC01 reduced hyperglycemia and type 2 diabetes mellitus by regulating glucose metabolism in the liver, protecting the islet β-cell mass, and restoring the gut microbiota. *L. plantarum* HAC01 may thus be an effective therapeutic agent for type 2 diabetes mellitus.	[114]
Human	The results of the study suggest that *L. plantarum* Y15 may serve as a potential agent for ameliorating type 2 diabetes through the regulation of the expression rate of genes responsible for pathways of increasing inflammation and insulin.	[115]
Human	The study assessed the effectiveness of probiotics on glycemic control in type 2 diabetes patients. 16-week probiotic supplementation (25 mL of beverage containing over 10^8^ CFU/mL of Lactobacillus, four times per day) resulted in no profitable effects on glycemic control, profile of lipids, as well as weight.	[108]

**Table 4:** Promising therapeutic effects of probiotic yogurt consumption in the inhibition of the growth of intestinal pathogens in humans.

No.	Effect	References
1	*Lactobacillus GG* significantly shortens the length of rotavirus diarrhea.	[130]
2	The duration of diarrhea in patients receiving *Lactobacillus GG* was 1.8 days, significantly shorter than those receiving *L. casei rhamnosus* (2.8 days) and *S. thermophilus* (2.6 days).	[131]
3	The study showed the beneficial effects of *B. bifidum* and *S. thermophilus* in preventing rotavirus diarrhea in infants. Their incidence was significantly lower in infants receiving probiotics (7%) than in the *placebo* group (35%).	[133]
4	Incidents of diarrhea in the children studied, thanks to the supply of *Lactobacillus GG*, occurred more than three times less often compared to the control group.	[135]
5	Supplementation with *Saccharomyces boulardi* reduced the risk of diarrhea in children by more than 10%.	[137]
6	The supply of *B. longum*, *L. rhamnosus*, and *L. plantarum* to patients aged 5 months to 16 years has an effect on reducing the number of stools per day.	[139]
7	The review showed that pathogenic microorganisms alter the homeostasis of the gut microbiota, leading to an increased risk of related diseases. The study demonstrated that probiotics inhibit these pathogens by stimulating epithelial barrier function, secreting anti-microbial components, competitively excluding pathogens by binding sites, and limiting their access to nutrients.	[140]
8	The article demonstrated that even a small number of genetic mutations or even a single-nucleotide variant in the microbial genome can significantly alter the pathogenic behavior of gut bacteria and affect host health. The study highlighted that probiotic consumption can significantly impede the proliferation of intestinal pathogenic bacteria.	[141]
9	The study showed that probiotics can interact with pathogens in foods and in the hosts. They are able to produce organic acids, bacteriocins, and hydrogen peroxide, which have an inhibitory effect on the growth of many pathogens in the gut.	[142]

## Data Availability

This review is based on the reported literature search, with no original data presented.

## References

[1] Salama H.H., Bhattacharya S., Singh J., Vyas A. (2022). Advancement of yogurt production technology. Advances in Dairy Microbial Products.

[2] Hadjimbei E., Botsaris G., Chrysostomou S. (2022). Beneficial effects of yoghurts and probiotic fermented milks and their functional food potential. Foods.

[3] Dimidi E., Cox S.R., Rossi M., Whelan K. (2019). Fermented foods: Definitions and characteristics, impact on the gut microbiota and effects on gastrointestinal health and disease. Nutrients.

[4] Marsh A.J., Hill C., Ross R.P., Cotter P.D. (2014). Fermented beverages with health-promoting potential: Past and future perspectives. Trends Food Sci. Technol..

[5] Gaba K., Anand S. (2023). Incorporation of probiotics and other functional ingredients in dairy fat-rich products: Benefits, challenges, and opportunities. Dairy.

[6] Ravyts F., De V.L., Leroy F. (2012). Bacterial diversity and functionalities in food fermentations. Eng. Life Sci..

[7] Markowiak P., Śliżewska K. (2018). The role of probiotics, prebiotics and synbiotics in animal nutrition. Gut Pathog..

[8] Ziaei R., Ghavami A., Khalesi S., Ghiasvand R., Mokari Y. (2021). The effect of probiotic fermented milk products on blood lipid concentrations: A systematic review and meta-analysis of randomized controlled trials. Nutr. Metab. Cardiovasc. Dis..

[9] Mozaffarian D., Hao T., Rimm E.B., Willett W.C., Hu F.B. (2011). Changes in diet and lifestyle and long-term weight gain in women and men. N. Engl. J. Med..

[10] Sen M. (2019). Role of probiotics in health and disease—A review. Int. J. Adv. Life Sci. Res..

[11] Vargas M.H., Del-Razo-Rodríguez R., López-García A., Lezana-Fernández J.L., Chávez J., Furuya M.E.Y., Marín-Santana J.C. (2017). Effect of oral glycine on the clinical, spirometric and inflammatory status in subjects with cystic fibrosis: A pilot randomized trial. BMC Pulm. Med..

[12] Albenberg L.G., Wu G.D. (2014). Diet and the intestinal microbiome: Associations, functions, and implications for health and disease. Gastroenterology.

[13] Wopereis H., Oozeer R., Knipping K., Belzer C., Knol J. (2014). The first thousand days—Intestinal microbiology of early life: Establishinga symbiosis. Pediatr. Allergy Immunol..

[14] Nishida A., Inoue R., Inatomi O., Bamba S., Naito Y., Andoh A. (2018). Gut microbiota in the pathogenesis of inflammatory bowel disease. Clin. J. Gastroenterol..

[15] Donohoe D.R., Garge N., Zhang X., Sun W., O’Connell T.M., Bunger M.K., Bultman S.J. (2011). The microbiome and butyrate regulate energy metabolism and autophagy in the mammalian colon. Cell Metab..

[16] Mayer E.A. (2011). Gut feelings: The emerging biology of gut-brain communication. Nat. Rev. Neurosci..

[17] Grenham S., Clarke G., Cryan J.F., Dinan T.G. (2011). Brain-gut-microbe communication in health and disease. Front. Physiol..

[18] Montiel-Castro A.J., Gonzalez-Cervantes R.M., Bravo-Ruiseco G., Pacheco-López G. (2013). The microbiota-gut-brain axis: Neurobehavioral correlates, health and sociality. Front. Integr. Neurosci..

[19] Dinan T.G., Stilling R.M., Stanton C., Cryan J.F. (2015). Collective unconscious: How gut microbes shape human behavior. J. Psychiatr. Res..

[20] de Vos W.M., de Vos E.A.J. (2012). Role of the intestinal microbiome in health and disease: From correlation to causation. Nutr. Rev..

[21] Lynch S.V., Pedersen O. (2016). The human intestinal microbiome in health and disease. N. Engl. J. Med..

[22] Rudzki L., Frank M., Szulc A., Gałęcka M., Szachta P., Barwinek D. (2012). Od jelit do depresji-rola zaburzeń ciągłości barieryjelitowej i następcza aktywacja układu immunologicznego w zapalnej hipotezie depresji. Neuropsychiatr. Neuropsychol..

[23] Duca F.A., Lam T.K.T. (2014). Gut microbiota, nutrient sensing and energy balance. Diabetes Obes. Metab..

[24] Mayer E.A., Savidge T., Shulman R.J. (2014). Brain-gut microbiome interactions and functional bowel disorders. Gastroenterology.

[25] Hill C., Guarner E., Reid G., Gibson G.R., Merenstein D.J., Pot B., Morelli L., Canani R.B., Flint H.J., Salminen S. (2014). Expert consensus document. The International Scientific Association for Probiotics and Prebiotics consensus statement on thescope and appropriate useof the term probiotic. Nat. Rev. Gastroenterol. Hepatol..

[26] Szajewska H. (2017). Probiotyki aktualny stan wiedzy i zalecenia dla praktyki klinicznej. Med. Prakt..

[27] Floch M.H., Walker W.A., Guandalini S., Hibberd P., Gorbach S., Surawicz C., Sanders M.E., Garcia-Tsao G., Quigley E.M.M., Isolauri E. (2008). Recommendations for probiotic use—2008. J. Clin. Gastroenterol..

[28] Szajewska H., Guarino A., Hojsak I., Indrio F., Kolacek S., Shamir R., Vandenplas Y., Weizman Z. (2014). European Society for Pediatrie Gastroenterology, Hepatology, and Nutrition. Use of probiotics for management of acute gastroenteritis: A position paper by the ESPGHAN Working Group for Probiotics and Prebiotics. J. Pediatr. Gastroenterol. Nutr..

[29] Riddle M.S., DuPont H.L., Connor B.A. (2016). ACG Clinical Guideline: Diagnosis, Treatment, and Prevention of Acute Diarrheal Infections in Adults. Am. J. Gastroenterol..

[30] Szajewska H., Canani R.B., Guarino A., Hojsak I., Indrio F., Kolacek S., Orel R., Shamir R., Vandenplas Y., van Goudoever J.B. (2016). ESPGHAN Working Group for Probiotics Prebiotics. Probiotics for the prevention of antibiotic- -associated diarrhea in children. J. Pediatr. Gastroenterol. Nutr..

[31] Szajewska H., Kołodziej M. (2005). Systematic review with meta-analysis: *Saccharomyces boulardii* in the prevention of antibiotic-associated diarrhea. Aliment. Pharmacol. Ther..

[32] Draper K., Ley C., Parsonnet J. (2017). Probiotic guidelines and physician practice: A cross-sectional survey and overview of the literature. Benef. Microbes.

[33] Szajewska H., Kołodziej M. (2015). Systematic review with meta-analysis: *Lactobacillus rhamnosus* GG in the prevention of antibiotic-associated diarrhoea in children and adults. Aliment. Pharmacol. Ther..

[34] Shen N.T., Maw A., Tmanova L.L., Pino A., Ancy K., Crawfors C.V., Simon M.S., Evans A.T. (2017). Timely Use of Probiotics in Hospitalized Adults Prevents *Clostridium difficile* Infection: A Systematic Review With Meta-Regression Analysis. Gastroenterology.

[35] Lau C.S., Chamberlain R.S. (2016). Probiotics are effective at preventing *Clostridium difficile*-associated diarrhea: A systematic review and meta-analysis. Int. J. Gen. Med..

[36] Whorwell P.I., Altringer L., Morel J., Bond Y., Chabonneau D., O’Mahony L., Kiely B., Shanahan F., Quigley E. (2006). Efficacy of an encapsulated probiotic *Bifidobacterium infantis* 35624 in women with irritable bowel syndrome. Am. J. Gastroenterol..

[37] Ducrotte P., Sawant P., Jayanthi V. (2012). Clinical trial: *Lactobacillus plantarum* 299v (DSM 9843) improves symptoms of irritable bowel syndrome. World J. Gastroenterol..

[38] Horvath A., Dziechciarz P., Szajewska H. (2011). Meta-analysis: *Lactobacillus rhamnosus* GG for abdominal pain-related functional gastrointestinal disorders in childhood. Aliment. Pharmacol. Ther..

[39] Guarner F., Sanders M.E., Szajewska H., Cohen H., Eliakim R., Herrera-deGuise C., Karakan T., Merenstein D., Piscoya A., Ramakrishna B. (2017). Probiotics and prebiotics. World Gastroenterology Organisation Global Guidelines.

[40] Harris K., Kassis A., Major G. (2012). Is the gut microbiota a new factor contributing to Obesity and its Metabolic disorders. J. Obes..

[41] Blaut M., Klaus S. (2012). Intestinal microbiota and Obesity. Handb. Exp. Pharmacol..

[42] Tumbaugh P., Ley R., Mahowald M. (2006). An Obesity-associated gut microbiome with increased capacity for Energy harvest. Nature.

[43] Ostrowska L. (2016). Mikrobiota jelitowa a zaburzenia metaboliczne i otyłość—Punkt widzenia internisty i dietetyka. Gastroenterol. Klin..

[44] Grudell A.B., Camilleri M. (2007). The role of peptide YY in integrative gut physiology and potential role in obesity. Curr. Opin. Endocrinol. Diabetes Obes..

[45] Amar J., Chabo C., Waget A., Klopp P., Vachoux C., Bermudez-Humaran L.G., Smirnova N., Berge M., Sulpice T., Lahtinen S. (2011). Intestinal mucosal adherence and translocation of commensal bacteria at the early onset oftype 2 diabetes: Molecular mechanism and probiotic treatment. EMBO Mol. Med..

[46] Cani P.D., Neyrinck A.M., Fava F., Knauf C., Burcelin R.G., Tuohy K.M., Gibson G.R., Delzenne N.M. (2007). Selective increases of bifidobacteria in gut microflora improve high-fat-diet-induced diabetes in mice through a mechanism associated with endotoxaemia. Diabetologia.

[47] Lee H.Y., Park J.H., Seok S.H., Baek M.W., Kim D.J., Lee K.E., Paek K.S., Lee Y., Park J.H. (2006). Human originated bacteria, *Lactobacillus rhamnosus* PL60, produce conjugated linoleic acid and show anti-obesity effects in diet-induced obese mice. Biochim. Biophys. Acta.

[48] Lee K., Paek K., Lee H.Y., Park J.H., Lee Y. (2007). Antiobesity effect of trans-10,cis-12-conjugated linoleic acid-producing *Lactobacillus plantarum* PL62 on diet-induced obese mice. J. Appl. Microbiol..

[49] Sato M., Uzu K., Yoshida T., Hamad E.M., Kawakami H., Matsuyama H., Abd El-Gawad I.A., Imaizumi K. (2008). Effects ofmilk fermented by *Lactobacillus gasseri* SBT2055 on adipocyte size in rates. Br. J. Nutr..

[50] Tomaro-Duchesneau C., Saha S., Malhotra M., Jones M.L., Labbe A., Rodes L., Kahouli I., Prakash S. (2014). Effect of orally administered L fermentum NCIMB 5221 on markers of metabolic syndrome: An in vivo analysis using ZDF rats. Appl. Microbiol. Biotechnol..

[51] Nerstedt A., Nilsson E.C., Ohlson K., Hakansson J., Svensson T., Lowenadler B., Svensson Y.K., Mahlapuu M. (2007). Administration of *Lactobacillus* evokes coordinated changes in the intestinal expression profile ofgenes regulating energy homeostasis and immune phenotype in mice. Br. J. Nutr..

[52] Kadooka Y., Sato M., Imaizumi K., Ogawa A., Ikuyama K., Akai Y., Okano M., Kagoshima M., Tsuchida T. (2010). Regulation of abdominal adiposity by probiotics (*Lactobacillus gasseri* SBT2055) in adults with obese tendencies in a randomized controlled trial. Eur. J. Clin. Nutr..

[53] Kadooka Y., Sato M., Ogawa A., Miyoshi M., Uenish H., Ogawa H., Ikuyama K., Kagoshima M., Ysuchida T. (2013). Effect of *Lactobacillus gasseri* SBT2055 in fermented milk on abdominal adiposity in adults in a randomised controll trial. Br. J. Nutr..

[54] Sanchez M., Darimont C., Drapeau V., Emady-Azar S., Lepage M., Rezzonico E., Ngom-Bru C., Berger B., Philippe L., Ammon-Zuffrey C. (2014). Effect of *Lactobacillus rhamnosus* CGMCC1.3724 supplementation on weight loss and maintenance in obese men and women. Br. J. Nutr..

[55] Babio N., Becerra-Tomas N., Martinez-Gonzalez M.A., Corella D., Estruch R., Ros E., Sayon-Orea C., Fito M., Serra-Majem L., Aros F. (2015). Consumption of yogurt, low-fat milk, and other low-fat dairy products is associated with lower risk of metabolic syndrome incidence in an elderly Mediterranean population. J. Nutr..

[56] Neyrinck A.M., Possemiers S., Druart C., Van de Wiele T., De Backer F., Cani P.D., Larondelle Y., Delzenne N.M. (2011). Prebiotic effects of wheat arabinoxylan related to the increase in *Bifidobacteria, Roseburia* and *Bacteroides/Prevotella* in diet-induced obese mice. PLoS ONE.

[57] Parnell J.A., Reimer R.A. (2012). Prebiotic fibres dose-dependently increase satiety hormones and alter Bacteroidetes and Firmicutes in lean and obese JCR: LA-cprats. Br. J. Nutr..

[58] Million M., Angelakis E., Paul M., Armougom F., Leibovici L., Raoult D. (2012). Comparative meta-analysis of the effect of *Lactobacillus* species on weight gain in humans and animals. Microb. Pathog..

[59] Angelakis E., Merhej V., Raoult D. (2013). Related actions of probiotics and antibiotics on gut microbiota and weight modification. Lancet Infect. Dis..

[60] Alvarez-Arrano V., Martin-Pelaez S. (2021). Effects of probiotics and synbiotics on weight loss in subjects with overweight or obesity: A systematic review. Nutrients.

[61] Musazadeh V., Zarezadeh M., Ghalichi F., Ahrabi S.S., Jamilian P., Jamilian P., Ghoreishi Z. (2022). Anti-obesity properties of probiotics; a considerable medical nutrition intervention: Findings from an umbrella meta-analysis. Eur. J. Pharmacol..

[62] Zhang Y., Yan T., Xu C., Yang H., Zhang T., Liu Y. (2021). Probiotics can further reduce waist circumference in adults with morbid obesity after bariatric surgery: A systematic review and meta-analysis of randomized controlled trials. Evid.-Based Complement. Altern. Med..

[63] Niloufar Rasaei N., Heidari M., Esmaeili F., Khosravi S., Baeeri M., Tabatabaei-Malazy O., Emamgholipour S. (2024). The effects of prebiotic, probiotic or synbiotic supplementation on overweight/obesity indicators: An umbrella review of the trials’ meta-analyses. Front. Endocrinol..

[64] Cani P.D., Neyrinck A.M., Maton N., Delzenne N.M. (2005). Oligo-fructose promotes satiety in rats fed a high-fat diet: Involvement of glucagon-like Peptide-1. Obes. Res..

[65] Cani P.D., Daubioul C.A., Reusens B., Remacle C., Catillon G., Delzenne N.M. (2005). Involvement of endogenous glucagon-like peptide-1 (7-36) amide on glycaemia-lowering effect of oligofructose in streptozotocin-treated rats. J. Endocrinol..

[66] Genta S., Cabrera W., Habib N., Pons J., Carillo I.M., Grau A., Sanchez A. (2009). Yacon syrup: Beneficial effects on obesity and insulin resistance in humans. Clin. Nutr..

[67] Cani P.D., Possemiers S., Van de Wiele T., Guiot Y., Everard A., Rottier O., Geurts L., Naslain D., Neyrinck A., Lambert D.M. (2009). Changes in gut microbiota control inflammation in obese mice through a mechanism involving GLP-2 driven improvement ofgut permeability. Gut.

[68] Shirvani-Rad S., Tabatabaei-Malazy O., Mohseni S., Hasani-Ranjbar S., Soroush A.-R., Hoseini-Tavassol Z., Ejtahed H.-S., Larijani B. (2021). Probiotics as a complementary therapy for management of obesity: A systematic review. Evid. Based Complement. Alternat. Med..

[69] Li Z., Li Y., Pan B., Wang X., Wu Y., Guo K., Yang M., Ma M., Qiao C., Yang K. (2022). The effects of oral probiotic supplementation in postmenopausal women with overweight and obesity: A systematic review and meta-analysis of randomized controlled trials. Probiotics Antimicrob. Proteins.

[70] Perna S., Ilyas Z., Giacosa A., Gasparri C., Peroni G., Faliva M.A., Rigon C., Naso M., Riva A., Petrangolini G. (2021). Is probiotic supplementation useful for the management of body weight and other anthropometric measures in adults affected by overweight and obesity with metabolic related diseases? A systematic review and meta-analysis. Nutrients.

[71] Mayta-Tovalino F., Diaz-Arocutipa C., Piscoya A., Hernandez A.V. (2023). Effects of probiotics on intermediate cardiovascular outcomes in patients with overweight or obesity: A systematic review and meta-analysis. J. Clin. Med..

[72] Hadi A., Alizadeh K., Hajianfar H., Mohammadi H., Miraghajani M. (2020). Efficacy of synbiotic supplementation in obesity treatment: A systematic review and meta-analysis of clinical trials. Crit. Rev. Food Sci. Nutr..

[73] Deng Y., Misselwitz B., Dai N., Fox M. (2015). Lactose intolerance in adults: Biological mechanism and dietary management. Nutrients.

[74] Oak S.J., Jha R. (2019). The effects of probiotics in lactose intolerance: A systematic review. Crit. Rev. Food Sci. Nutr..

[75] Masoumi S.J., Mehrabani D., Saberifiroozi M., Fattahi M.R., Moradi F., Najafi M. (2021). The effect of yogurt fortified with *Lactobacillus acidophilus* and *Bifidobacterium* sp. probiotic in patients with lactose intolerance. Food Sci. Nutr..

[76] Savaiano D.A. (2014). Lactose digestion from yogurt: Mechanism and relevance. Am. J. Clin. Nutr..

[77] Heyman M.B. (2006). Committee on Nutrition. Lactose intolerance in infants, children, and adolescents. Pediatrics.

[78] Sahi T., Isokoski M., Jussila J., Launiala K., Pyorala K. (1973). Recessive inheritance of adult-type lactose malabsorption. Lancet.

[79] Enattah N.S., Sahi T., Savilahti E., Terwilliger J.D., Peltonen L., Jarvela I. (2002). Identification of a variant associated with adult-type hypolactasia. Nat. Genet..

[80] Adolfsson O., Meydani S.N., Russell R.M. (2004). Yogurt and gut function. Am. J. Clin. Nutr..

[81] Thoreux K., Balas D., Bouley C., Senegas-Balas F. (1998). Diet supplemented with yoghurt or milk fermented by *Lactobacillus casei* DN-114 001 stimulates growth and brush-border enzyme activities in mouse small intestine. Digestion.

[82] Ahn S., Kim M.S., Park D.G., Han B.K., Kim Y.J. (2023). Effects of probiotics administration on lactose intolerance in adulthood: A meta-analysis. J. Dairy Sci..

[83] Ibrahim S.A., Gyawali R., Awaisheh S.S., Krastanov A.I. (2021). Fermented foods and probiotics: An approach to lactose intolerance. J. Dairy Res..

[84] Aguilera G., Cárcamo C., Soto-Alarcón S., Gotteland M. (2021). Improvement in lactose tolerance in hypolactasic subjects consuming ice creams with high or low concentrations of *Bifidobacterium bifidum* 900791. Foods.

[85] Gingold-Belfer R., Levy S., Layfer O., Pakanaev L., Niv Y., Dickman R., Perets T. (2020). Use of a novel probiotic formulation to alleviate lactose intolerance symptoms—A pilot study. Probiotics Antimicrob. Proteins.

[86] Vitellio P., Celano G., Bonfrate L., Gobbetti M., Portincasa P., De Angelis M. (2019). Effects of *Bifidobacterium longum* and *Lactoibacillus rhamnosus* on Gut Microbiota in Patients with Lactose Intolerance and Persisting Functional Gastrointestinal Symptoms: A Randomised, Double-Blind, Cross-Over Study. Nutrients.

[87] Leis R., de Castro M., de Lamas C., Couce M.L. (2020). Effects of Prebiotic and Probiotic Supplementation on Lactase Deficiency and Lactose Intolerance: A Systematic Review of Controlled Trials. Nutrients.

[88] Gijsbers L., Ding E.L., Malik V.S., De Goede J., Geleijnse J.M., Soedamah-Muthu S.S. (2016). Consumption of dairy foods and diabetes incidence: A dose-response meta-analysis of observational studies. Am. J. Clin. Nutr..

[89] Ivey K.L., Lewis J.R., Hodgson J.M., Zhu K., Dhaliwal S.S., Thompson P.L., Richard P. (2011). Association between yogurt, milk, and cheese consumption and common carotid artery intima-media thickness and cardiovascular disease risk factors in elderly women. Am. J. Clin. Nutr..

[90] Margolis K.L., Wei F., de Boer I.H., Howard B.V., Liu S., Manson J.E., Mossavar-Rahmani Y., Phillips L.S., Shikany J.M., Tinker L.F. (2011). A diet high in low-fat dairy products lowers diabetes risk in postmenopausal women. J. Nutr..

[91] Liu J.E., Zhang Y., Zhang J., Dong P.L., Chen M., Duan Z.P. (2010). Probiotic yogurt effects on intestinal flora of patients with chronic liver disease. Nurs. Res..

[92] Salas-Salvado J., Guasch-Ferre M., Diaz-López A., Babio N. (2017). Yogurt and diabetes: Overview of recent observational studies. J. Nutr..

[93] www.stat.gov.pl.

[94] Pokrzywnicka P., Gumprecht J. (2016). “Mikrobiota i jej związek z cukrzycą typu 2 i otyłością” Szpital Miejski w Zabrzu, Katedra i Klinika Chorób Wewnętrznych, Diabetologii i Nefrologii, Śląski Uniwersytet Medyczny w Zabrzu. Diabetol. Prakt..

[95] Konturek S. (2013). Fizjologia Człowieka Podręcznik dla Studentów Medycyny.

[96] Stawerska R., Łupińska A., Bieniek E., Lewiński A. (2021). Insulinooporność i stan przedcukrzycowy. Lekarz POZ.

[97] Sadagurski M., White M.F. (2013). Integrating metabolism and longevity through insulin and IGF1 signaling. Endocrinol. Metab. Clin. N. Am..

[98] Nowosad K. (2021). Rola diety i Stylu Życia w Leczeniu Insulinooporności.

[99] Gołąbek K.D., Regulska-Ilow B. (2019). Dietary support in insulin resistance: An overview of current scientific reports. Adv. Clin. Exp. Med..

[100] Ostrowska L., Witczak K., Adamska E. (2012). Czy istnieją środowiskowe uwarunkowania insulinooporności?. Forum Zaburzeń Metab..

[101] Noormohammadi M., Ghorbani Z., Löber U., Mahdavi-Roshan M., Bartolomaeus T.U.P., Kazemi A., Shoaibinobarian N., Forslund S.K. (2023). The effect of probiotic and synbiotic supplementation on appetite-regulating hormones and desire to eat: A systematic review and meta-analysis of clinical trials. Pharmacol. Res..

[102] Stachowicz M., Janas-Kozik M., Olszanecka-Glinianowicz M., Chudek J. (2013). Rola leptyny w zaburzeniach odżywiania się—Współczesne poglądy. Psychiatr. Pol..

[103] Kim S.W., Park K.Y., Kim B., Kim E., Hyun C.K. (2013). *Lactobacillus rhamnosus* GG improves insulin sensitivity and reduces adiposity in high-fat diet-fed mice through enhancement of adiponectin production. Biochem. Biophys. Res. Commun..

[104] Raji Lahiji M., Zarrati M., Najafi S., Yazdani B., Cheshmazar E., Razmpoosh E., Janani L., Lahiji M.R., Shidfar F. (2021). Effects of synbiotic supplementation on serum adiponectin and inflammation status of overweight and obese breast cancer survivors: A randomized, triple-blind, placebo-controlled trial. Support. Care Cancer.

[105] Hulston C.J., Churnside A.A., Venables M.C. (2015). Probiotic supplementation prevents high-fat, overfeeding-induced insulin resistance in human subjects. Br. J. Nutr..

[106] Elbere I., Silamikelis I., Dindune I.I., Kalnina I., Ustinova M., Zaharenko L., Silamikele L., Rovite V., Gudra D., Konrade I. (2020). Baseline gut microbiome composition predicts metformin therapy short-term efficacy in newly diagnosed type 2 diabetes patients. PLoS ONE.

[107] Zhang Y., Guo X., Guo J., He Q., Li H., Song Y., Zhang H. (2014). *Lactobacillus casei* reduces susceptibility to type 2 diabetes via microbiota-mediated body chloride ion influx. Sci. Rep..

[108] Peng X., Xian H., Ge N., Hou L., Tang T., Xie D., Gao L., Yue J. (2024). Effect of probiotics on glycemic control and lipid profiles in patients with type 2 diabetes mellitus: Arandomized, double blind, controlled trial. Front. Endocrinol..

[109] Li S., Liu Z., Zhang Q., Su D., Wang P., Li Y., Shi W., Zhang Q. (2024). The Antidiabetic Potential of Probiotics: A Review. Nutrients.

[110] Shen X., Ma C., Yang Y., Liu X., Wang B., Wang Y., Zhang G., Bian X., Zhang N. (2024). The Role and Mechanism of Probiotics Supplementation in Blood Glucose Regulation: A Review. Foods.

[111] Toejing P., Khampithum N., Sirilun S., Chaiyasut C., Lailerd N. (2021). Influence of *Lactobacillus paracasei* HII01 Supplementation on Glycemia and Inflammatory Biomarkers in Type 2 Diabetes: A Randomized Clinical Trial. Foods.

[112] Gu Y.X., Chen H.R., Li X., Li D., Sun Y., Yang L., Ma Y., Chan E.C.Y. (2023). *Lactobacillus paracasei* IMC 502 ameliorates type 2 diabetes by mediating gut microbiota-SCFA-hormone/inflammation pathway in mice. J. Sci. Food Agric..

[113] Wang Y.M., Dilidaxi D., Wu Y.C., Sailike J., Sun X., Nabi X.H. (2020). Composite probiotics alleviate type 2 diabetes by regulating intestinal microbiota and inducing GLP-1 secretion in db/db mice. Biomed. Pharmacother..

[114] Lee Y.S., Lee D., Park G.S., Ko S.H., Park J., Lee Y.K., Kang J. (2021). *Lactobacillus plantarum* HAC01 ameliorates type 2 diabetes in high-fat diet and streptozotocin-induced diabetic mice in association with modulating the gut microbiota. Food Funct..

[115] Liu Y., Zheng S.J., Cui J.L., Guo T.T., Zhang J.T. (2022). Lactiplantibacillus plantarum Y15 alleviate type 2 diabetes in mice via modulating gut microbiota and regulating NF-κB and insulin signaling pathway. Braz. J. Microbiol..

[116] Pegah A., Abbasi-Oshaghi E., Khodadadi I., Mirzaei F., Tayebinai H. (2021). Probiotic and resveratrol normalize GLP-1 levels and oxidative stress in the intestine of diabetic rats. Metab. Open.

[117] Naseri K., Saadati S., Ghaemi F., Ashtary-Larky D., Asbaghi O., Sadeghi A., Afrisham R., de Courten B. (2023). The effects of probiotic and synbiotic supplementation on inflammation, oxidative stress, and circulating adiponectin and leptin concentration in subjects with prediabetes and type 2 diabetes mellitus: A GRADE-assessed systematic review, meta-analysis, and meta-regression of randomized clinical trials. Eur. J. Nutr..

[118] Zhao J.Y., Wang L.H., Cheng S.S., Zhang Y., Yang M., Fang R.X., Li H.X., Man C.X., Jiang Y.J. (2022). A Potential Synbiotic Strategy for the Prevention of Type 2 Diabetes: *Lactobacillus paracasei* JY062 and Exopolysaccharide Isolated from *Lactobacillus plantarum* JY039. Nutrients.

[119] Xiao R., Wang L.L., Tian P.J., Jin X., Zhao J.X., Zhang H., Wang G., Zhu M.M. (2023). The Effect of Probiotic Supplementation on Glucolipid Metabolism in Patients with Type 2 Diabetes: A Systematic Review and Meta-Analysis. Nutrients.

[120] Rodrigues R.R., Gurung M., Li Z.P., García-Jaramillo M., Greer R., Gaulke C., Bauchinger F., You H., Pederson J.W., Vasquez-Perez S. (2021). Transkingdom interactions between Lactobacilli and hepatic mitochondria attenuate western diet-induced diabetes. Nat. Commun..

[121] Jiang H.R., Cai M.M., Shen B.Y., Wang Q., Zhang T.C., Zhou X. (2022). Synbiotics and Gut Microbiota: New Perspectives in the Treatment of Type 2 Diabetes Mellitus. Foods.

[122] Gulnaz A., Nadeem J., Han J.H., Lew L.C., Son J.D., Park Y.H., Rather I.A., Hor Y.Y. (2021). *Lactobacillus* sps in Reducing the Risk of Diabetes in High-Fat Diet-Induced Diabetic Mice by Modulating the Gut Microbiome and Inhibiting Key Digestive Enzymes Associated with Diabetes. Biology.

[123] Zhou X.R., Shang G.S., Tan Q., He Q., Tan X.Y., Park K.Y., Zhao X. (2021). Effect of *Lactobacillus fermentum* TKSN041 on improving streptozotocin-induced type 2 diabetes in rats. Food Funct..

[124] Archer A.C., Muthukumar S.P., Halami P.M. (2021). *Lactobacillus fermentum* MCC2759 and MCC2760 Alleviate Inflammation and Intestinal Function in High-Fat Diet-Fed and Streptozotocin-Induced Diabetic Rats. Probiotics Antimicrob. Proteins.

[125] Koczarski M. (2020). The Effect of Probiotic Supplementation on Glycemic Control in Women with Gestational Diabetes Mellitus A Systematic Review. Top. Clin. Nutr..

[126] Wu L., Gao Y., Su Y., Li J., Ren W.C., Wang Q.H., Kuang H.X. (2022). Probiotics with anti-type 2 diabetes mellitus properties: Targets of polysaccharides from traditional Chinese medicine. Chin. J. Nat. Med..

[127] Maciorkowska E., Ryszczuk E., Kaczmarski M. (2010). Rola probiotyków i prebiotyków w apoptozie przewodu pokarmowego. Przegląd Gastroenterol..

[128] Ignyś I., Piątkowska P., Cichy W. (2008). Probiotyki i prebiotyki w żywieniu i leczeniu dzieci. Ped. Pol..

[129] Cichy W., Ignyś I., Piątkowska P. (2006). Współczesne poglądy na profilaktyczne i terapeutyczne zastosowanie probiotyków u dzieci. Świat Med. Farm..

[130] Isolauri E., Juntunen M., Rautanen T., Sillanaukee P., Koivula T. (1991). A human *Lactobacillus* strain (*Lactobacillus casei* sp. Strain GG) promotes recovery from acute diarrhoea in children. Pediatrics.

[131] Majamaa H., Isolauri E., Saxelin M., Vesikari T. (1995). Lactic acid bacteria in the treatment of acute rotavirus gastroenteritis. J. Pediatr. Gastroenterol. Nutr..

[132] Shornikova A.V., Isolauri E., Casas I.A., Vesikari T. (1996). *Lactobacillus reuteri* effectively colonises GI tract and shortens acute diarrhoea in children. J. Pediatr. Gastroenterol. Nutr..

[133] Saavedra J.M., Bauman N.A., Perman J.A., Yolken R.H., Saavedra J.M., Bauman N.A., Oung I. (1994). Feeding of *Bifidobacterium bifidum* and *Streptococcus thermophilus* to infants in hospital for prevention of diarrhoea and sheeding of rotavirus. Lancet.

[134] Ruszczyński M., Szajewska H. (2008). Probiotyki w zapobieganiu biegunce związanej ze stosowaniem antybiotyków—Aktualizacja metaanalizy badań z randomizacją. Pediatr. Współcz..

[135] Arvola T., Laiho K., Torkkeli S., Mykkanen H., Salminen S., Maunula L., Isolauri E. (1999). Prophylactic *Lactobacillus* GG reduces antibiotic-assosiated diarrhea in children with respiratory infections: A randomized study. Pediatrics.

[136] Vanderhoof J.A., Whitnej D.B., Antonson D.L., Hanner T.L., Lupo J.V., Young R.J. (1999). *Lactobacillus* GG in the prevention of antibiotic-associated diarrhea in children. J. Pediatr..

[137] Piątkowska P., Ignyś I., Roszak D., Cichy W. (2007). Probiotyki a antybiotykoterapia u dzieci. Świat Med. Farm..

[138] Kotowska M., Albrecht P., Szajewska H. (2005). *Saccharomyces boulardii* in the prevention of antibiotic-associated diarrhoea in children: A randomized double-blind placebo-controlled trial. Aliment. Pharmacol. Ther..

[139] Szamański H., Armańska M., Kowalska-Duplaga K., Szajewska H. (2008). *Bifidobacterium longum* PL03, *Lactobacillus rhamnosus* KL53A and *Lactobacillus plantarum* PL02 in the prevention of antibiotic-associated diarrhoea in children: A randomized controlled pilot trial. Digestion.

[140] Chandrasekaran P., Weiskirchen S., Weiskirchen R. (2024). Effects of Probiotics on Gut Microbiota: An Overview. Int. J. Mol. Sci..

[141] Ma C., Zhang C., Chen D., Jiang S., Shen S., Huo D., Huang S., Zhai Q., Zhang J. (2021). Probiotic consumption influences universal adaptive mutations in indigenous human and mouse gut microbiota. Commun. Biol..

[142] Khaneghah A.M., Abhari K., Eş I., Soares M.B., Oliveira R.B.A., Hosseini H., Rezaei M., Balthazar C.F., Silva R., Cruz A.G. (2020). Interactions between probiotics and pathogenic microorganisms in hosts and foods: A review. Trends Food Sci. Technol..

[143] D’Argenio V., Salvatore F. (2015). The role of the gut microbiome in the healthy adult status. Clin. Chim. Acta.

[144] Sun J., Chang E.B. (2014). Exploring gut microbes in human health and disease: Pushing the envelope. Genes Dis..

[145] Javanmard A., Ashtari S., Sabet B., Davoodi S.H., Rostami-Nejad M., Akbari M.E., Niaz A., Mortazavian A.M. (2018). Probiotics and their role in gastrointestinal cancer prevention and treatment: An overview. Gastroenterol. Hepatol. Bed Bench.

[146] Wang L., Zhou J., Xin Y., Geng C., Tian Z., Yu X., Zibin Y., Xinjuan Y., Quanjiang D. (2016). Bacterial overgrowth and diversification of microbiota in gastric cancer. Eur. J. Gastroenterol. Hepatol..

[147] Dai Z., Zhang J., Wu Q., Chen J., Liu J., Wang L., Chen C., Xu J., Zhang H., Shi C. (2018). The role of microbiota in the development of colorectal cancer. Int. J. Cancer.

[148] Kaźmierczak-Siedlecka K., Ruszkowski J., Folwarski M., Obara K., Makarewicz W., Lebiedzińska A. (2019). Zastosowanie fitoterapii w leczeniu wrzodziejącego zapalenia jelita grubego. Bromatol. Chem. Toksykol..

[149] Koziński K., Dobrzyń A. (2013). Szlak sygnałowy Wnt i jego rola w regulacji metabolizmu komórki. Postępy Hig. Med. Doświadczalnej.

[150] Kuśmierska A., Fol M. (2014). Właściwości immunomodulacyjne i terapeutyczne drobnoustrojów probiotycznych. Probl. Hig. Epidemiol..

[151] Yu A., Li L. (2016). The Potential Role of Probiotics in Cancer Prevention and Treatment. Nutr. Cancer.

[152] Mojka K. (2014). Probiotyki, prebiotyki i synbiotyki—Charakterystyka i funkcje. Probl. Hig. Epidemiol..

[153] Holscher H.D. (2017). Dietary fiber and prebiotics and the gastrointestinal microbiota. Gut Microbes.

[154] Siepsiak M., Połom A., Adrych K. (2015). Profilaktyka raka jelita grubego. Farm Współ..

[155] Rothwell P.M., Wilson M., Elwin C.E. (2010). Long-term effect of aspirin on colorectal cancer incidence and mortality: 20-year follow-up of five randomised trials. Lancet.

[156] Ambalam P., Raman M., Purama R.K., Doble M. (2016). Probiotics, Prebiotics and Colorectal Cancer Prevention. Best Pract. Res. Clin. Gastroenterol..

[157] O’Mahony L., Feeney M., O’Halloran S., Murphy L., Kiely B., Fitzgibbon J., Lee G., O’Sullivan L.G., Shanahan F., Collins J.K. (2001). Probiotic impact on microbial flora, inflammation and tumour development in IL-10 knockout mice. Aliment. Pharmacol. Ther..

[158] Kozieł N., Kukier E., Kwiatek K. (2019). *Clostridium perfringens*—Znaczenie epidemiologiczne i diagnostyka zachorowań. Med. Weter..

[159] Horie H., Zeisig M., Hirayama K., Midtvedt T., Moller L., Rafter J. (2003). Probiotic mixture decreases DNA adduct formation in colonic epithelium induced by the food mutagen 2-amino-9H-pyrido[2,3-b]indole in a human-flora associated mouse model. Eur. J. Cancer Prev..

[160] Bertkova I., Hijova E., Chmelarova A., Mojzisova G., Petrasova D., Strojny L., Bomba A., Zitnan R. (2010). The effect of probiotic microorganisms and bioactive compounds on chemically induced carcinogenesis in rats. Neoplasma.

[161] Ma E.L., Choi Y.J., Choi J., Pothoulakis C., Rhee S.H., Im E. (2010). The anticancer effect of probiotic Bacillus polyfermenticus on human colon cancer cells is mediated through ErbB2 and ErbB3 inhibition. Int. J. Cancer.

[162] Bertucci F., Borie N., Ginestier C., Groulet A., Charafe-Jauffret E., Adélaïde J., Geneix J., Bachelart L., Finetti P., Koki A. (2004). Identification and validation of an ERBB2 gene expression signature in breast cancers. Oncogene.

[163] Hatakka K., Holma R., El-Nezami H., Suomalainen T., Kuisma M., Saxelin M., Poussa T., Mykkanen H., Korpela R. (2008). The influence of *Lactobacillus rhamnosus* LC705 together with Propionibacterium freudenreichii ssp. shermanii JS on potentially carcinogenic bacterial activity in human colon. Int. J. Food Microbiol..

[164] Jacouton E., Chain F., Sokol H., Langella P., Bermudez-Humaran G.L. (2017). Probiotic Strain *Lactobacillus casei* BL23 Prevents Colitis-Associated Colorectal Cancer. Front. Immunol..

[165] Deng X., Yang J., Zhang Y., Chen X., Wang C., Suo H., Song J. (2023). An Update on the Pivotal Roles of Probiotics, Their Components, and Metabolites in Preventing Colon Cancer. Foods.

[166] Budu O., Banciu C., Pinzaru I., Sarău C., Lighezan D., Șoica C., Dehelean C., Drăghici G., Dolghi A., Prodea A. (2022). A combination of two probiotics, *Lactobacillus sporogenes* and *Clostridium butyricum*, inhibits Colon Cancer Development: An In Vitro Study. Microorganisms.

[167] Aindelis G., Tiptiri-Kourpeti A., Lampri E., Spyridopoulou K., Lamprianidou E., Kotsianidis I., Ypsilantis P., Pappa A., Chlichlia K. (2020). Immune responses raised in an experimental colon carcinoma model following oral administration of *Lactobacillus casei*. Cancers.

[168] Wang T., Zhang L., Wang P., Liu Y., Wang G., Shan Y., Yi Y., Zhou Y., Liu B., Wang X. (2022). *Lactobacillus coryniformis* MXJ32 administration ameliorates azoxymethane/dextran sulfate sodium-induced colitis-associated colorectal cancer via reshaping intestinal microenvironment and alleviating inflammatory response. Eur. J. Nutr..

[169] Shang F., Jiang X., Wang H., Chen S., Wang X., Liu Y., Guo S., Li D., Yu W., Zhao Z. (2020). The inhibitory effects of probiotics on colon cancer cells: In vitro and in vivo studies. J. Gastrointest. Oncol..

[170] Janelle A.C., Raad Z.G., Joshua M.U., Perez-Chanona E., Sha W., Tomkovich S., Mühlbauer M., Fodor A.A., Jobin C. (2013). VSL#3 probiotic modifies mucosal microbial composition but does not reduce colitis-associated colorectal cancer. Sci. Rep..

[171] Sunkata R., Herring J., Walker L.T., Verghese M. (2014). Chemopreventive Potential of Probiotics and Prebiotics. Food Nutr. Sci..

[172] Wang T., Wang P., Yin L., Wang X., Shan Y., Yi Y., Zhou Y., Liu B., Wang X., Lü X. (2023). Dietary *Lactiplantibacillus plantarum* KX041 attenuates colitis-associated tumorigenesis and modulates gut microbiota. Food Sci. Hum. Wellness.

[173] Czerwionka-Szaflarska M., Kuczyńska R., Mierzwa G., Bała G., Murawska S. (2006). Ocena wpływu bakterii probiotycznych na tolerancję terapii eradykacyjnej zakażeń *Helicobacterpylori* u dzieci i młodzieży. Ped. Pol..

[174] Szajewska H. (2005). Rola probiotyków w zapobieganiu i leczeniu chorób przewodu pokarmowego. Pediatr. Współcz..

[175] Sykora J., Valeckova K., Amlerova J., Siala K., Dedek P., Watkins S., Varvarovska J., Stozicky F., Pazdiora P., Schwarz J. (2005). Effects of a specially de- signed fermented milk product containing probiotic *Lactobacillus casei* DN-114 001 and the eradication of *H. pylori* in children: A pro- spective randomized double-blind study. J. Clin. Gastroenterol..

[176] Nista E.C., Candelli M., Cremonini F., Cazzato I.A., Zocco M.A., Franceschi F., Cammarota G., Gasbarrini G., Gasbarrini A. (2004). *Bacillus clausi* therapy to reduce side-effects of anti-*Helicobacter pylori* treatment: Randomiz- ded, double-blind, placebo controlled trial. Aliment. Pharmacol. Ther..

[177] Malfertheiner P., Megraud F., O’Morain C.A., Gisbert J.P., Kuipers E.J., Axon A.T., Bazzoli F., Gasbarrini A., Artherton J., Graham D.Y. (2017). European Helicobacter and microbiota study group and concensus panel. Management of Helicobacter pylori infection—The Mastricht V/Florence Consensus Report. Gut.

[178] Ford A.C., Quigley E.M.M., Lacy B.E., Lembo A., Saito Y., Schiller L.R., Soffer E., Spiegel B., Moayyedi P. (2014). Efficacy of prebiotics, probiotics, and synbiotics in irritable bowel syndrome and chronic idiopathic constipation: Systematic review and meta-analysis. Am. J. Gastroenterol..

[179] Hungin A.P.S., Mitchell C.R., Whorwell P., Mulligan C., Cole O., Agreus L., Fracasso P., Lionis C., Mendive J., Philippart de Foy J.-M. (2018). Systematic review: Probiotics in the management of lower gastrointestinal symptoms—An updated evidence-based International consensus. Aliment. Pharmacol. Ther..

[180] Strzelec B., Stuła M., Chmielewski P., Taboła R. (2018). Profilaktyka i leczenie raka żołądka—Aktualny problem interdyscyplinarny. Pol. Nurs..

[181] Yu G., Hu N., Wang L., Wang C., Han X.Y., Humpry M., Ravel J., Abnet C.C., Taylor P.R., Goldstein A.M. (2017). Gastric microbiota features associated with cancer risk factors and clinical outcomes: A pilot study in gastric cardia cancer patients from Shanxi, China. Int. J. Cancer.

[182] Dyrla P., Gil J., Wojtuń S., Korszun K., Kasińska E., Mackiewicz A. (2015). Zakażenie *Helicobacter pylori*. Diagnostyka i leczenie. Pediatr. Med. Rodz..

[183] Castano-Rodriquez N., Goh K.L., Fock K.M., Hazel M.M., Nadeem O.K. (2017). Dysbiosis of the microbiome in gastric cancer. Sci. Rep..

[184] Namkin K., Zardast M., Basirinejad F. (2016). *Saccharomyces boulardii* in *Helicobacter pylori* eradication in children: A randomized trial from Iran. Iran. J. Pediatr..

[185] Bin Z., Ya-Zheng X., Zhao-Hui D., Bo C., Li-Rong J., Vandenplas Y. (2015). The efficiency of *Saccharomyces boulardii* CNCM I-745 in addition to standard *Helicobacter pylori* eradication treatment in children. Pediatr. Gastroenterol. Hepatol. Nutr..

[186] Szajewska H., Horvath A., Kołodziej M. (2015). Systematic review and meta-analysis: *Saccharomyces boulardii* supplementation and eradication of *Helicobacter pylori* infection. Aliment. Pharmacol. Ther..

[187] Tong J.L., Ran Z.H., Shen J., Zhang C.X., Xiao S.D. (2007). Meta-analysis: The effect of supplementation with probiotics on eradication rates and adverse events during *Helicobacter pylori* eradication therapy. Aliment. Pharmacol. Ther..

[188] Eslami M., Yousefi B., Kokhaei P., Moghadas A.J., Moghadam B.S., Arabkari V., Niazi Z. (2019). Are Probiotics Useful for Therapy of *Helicobacter pylori* Diseases?. Comp. Immunol. Microbiol. Infect. Dis..

